# Transcranial photoacoustic imaging of indocyanine green J-aggregates reveals cerebral signal changes in lipopolysaccharide-induced systemic inflammation

**DOI:** 10.1016/j.pacs.2026.100885

**Published:** 2026-09-22

**Authors:** Filip J. Bodera, Elizabeth Berndl, Shrishti Singh, Anthony Tay, Greeshma Tarimala, Samuel K. Jensen, Parag V. Chitnis, Remi Veneziano, Mark J. McVey, Michael C. Kolios

**Affiliations:** a Department of Physics, Toronto Metropolitan University, Toronto, Canada; b Institute of Biomedical Engineering, Science and Technology, Li Ka Shing Knowledge Institute, Keenan Research Centre, St. Michael’s Hospital, Toronto, Canada; c Translational Medicine Program, The Hospital for Sick Children, Toronto, Canada; d Department of Bioengineering, George Mason University, Fairfax, VA, 22030, United States; e Department of Chemical, Biochemical, and Environmental Engineering, University of Maryland, Baltimore County, United States; f The Center for Adaptive Systems of Brain-Body Interactions, George Mason University, Fairfax, VA, 22030, United States; g Department of Anesthesiology and Pain Medicine, University of Toronto, Toronto, ON, Canada; h Department of Physiology, University of Toronto, Toronto, ON, Canada; i Department of Anesthesia and Pain Medicine, The Hospital for Sick Children, Toronto, ON, Canada

**Keywords:** Transcranial, Lipopolysaccharide, Indocyanine, J-aggregates, Inflammation, Photoacoustic, Contrast, Cerebral

## Abstract

**Background:**

Systemic inflammation associated with surgery, infections, and trauma can damage organs, including the brain, and contribute to stroke and cognitive decline. Rapid assessment of inflammation-associated cerebrovascular changes remains difficult with existing neuroimaging modalities, particularly in settings requiring repeated or rapid measurements. Contrast-enhanced photoacoustic (PA) imaging may provide a research approach for monitoring cerebral vasculature.

**Objective:**

This pilot study investigated intracranial changes in dynamic PA signals of a novel photoacoustic imaging (PAI) tracer, non-encapsulated indocyanine green J-aggregates (J-aggs), in a model of lipopolysaccharide (LPS)-induced inflammation. This work is a pilot study to assess the potential of J-aggs for detecting systemic inflammation related changes in brain PAI signals.

**Methods:**

Systemic inflammation was induced in Sprague-Dawley rats with intraperitoneal (IP) LPS (3 mg/kg, n = 6) while sham rats received equal volume IP saline injections (n = 5). Transcranial PAI was performed before IP injections, 2-hours, and 4-hours after IP injections. Within the cerebral region of interest, P90 was defined as the mean PA signal intensity of voxels above the 90th-percentile intensity threshold, corresponding to the upper 10% of pixel values; group×time interactions were assessed using a linear mixed-effects model.

**Results:**

Intravenous J-aggs produced sustained PAI enhancement in sham rat brains (ΔP90(%) ~70% at 4-hours) whereas LPS-treated rats showed attenuated and more variable enhancement (ΔP90(%) ~30% at 4 h; group×time p = 0.0262). Reduced cerebral PA signal in LPS-treated rats may reflect altered J-aggs circulation or cerebral delivery, inflammation-related changes in cerebral blood volume, oxygenation, perfusion, or a combination of these factors.

**Conclusion:**

Because imaging was performed at a single wavelength of 890 nm, P90 represents a composite photoacoustic signal arising from J-aggs and endogenous hemoglobin; therefore, the attenuated response in LPS-treated rats may reflect altered J-aggs circulation or cerebral delivery, inflammation-related changes in cerebral blood volume or oxygenation, or a combination of these mechanisms.

## 1 Introduction

Infections, trauma, or surgery can trigger systemic inflammatory responses. Circulating inflammatory cytokines increase endothelial cell interaction and adhesion with immune cells. Endothelial-immune cell interactions lead to microvascular permeability and tissue damage, which, in part, contribute to multi-organ dysfunction [1,2]. Though inflammatory responses eliminate harmful infectious stimuli [3], dysregulation can lead to sepsis, multi-organ dysfunction, and increased morbidity and mortality [4,5]. Among organs that can be affected by systemic inflammation, the brain is particularly vulnerable [6]. Circulating cytokines and activated immune cells can alter blood–brain barrier integrity [7], disrupt cerebral autoregulation [8], and trigger microglial activation [9]. These processes can influence cerebral hemodynamics [10], leading to transient or lasting changes in brain function. Assaying cytokines at the bedside to measure inflammation can take hours to acquire information using antibody-based detection methods such as enzyme linked immunosorbent assays.

Faster (indirect) measurements of inflammation are possible with imaging. Current imaging modalities used to assess inflammation, including magnetic resonance imaging (MRI), positron emission tomography (PET), computed tomography (CT), and single-photon emission computed tomography (SPECT) provide valuable structural or molecular information but are limited in their ability to offer rapid, bedside assessment [[11], [12], [13]]. PET and SPECT offer molecular sensitivity through radiotracer-labeled ligands targeting inflammation-specific biomarkers such as translocator protein or vascular cell adhesion molecule 1 (VCAM-1), but these approaches require radioactive tracers, specialized infrastructure, and are not suited for rapid monitoring [[14], [15], [16]]. CT is commonly used clinically but lacks molecular specificity and requires exposure to ionizing radiation [11]. A common limitation across all clinical modalities is their inability to provide rapid measurements that could support changes in clinical management, such as during surgery or when managing acute trauma. Further, due to their size or special requirements, these modalities cannot be used intraoperatively. These limitations motivate investigation of imaging approaches that could eventually support rapid, repeated assessment of inflammation-associated cerebrovascular changes which may be linked to neuroinflammation-mediated morbidity, such as strokes or cognitive decline [17].

One model commonly used method to simulate infection or trauma induces a systemic inflammatory response by administering an intraperitoneal (IP) injection of lipopolysaccharide (LPS), a component of the complex pathogen-associated molecular pattern released by Gram-negative bacteria [18]. LPS causes a systemic inflammatory response through several pathways, including the toll-like receptor 4 (TLR-4) pathway [19], the nuclear kappa beta pathway [20], and the Janus kinase/signal transducer and activator of transcription (JAK-STAT) pathway [21]. Activation of these inflammatory pathways results in increased cytokine production [20], reactive oxygen species [22], and inflammasome activation [19].

Photoacoustic imaging (PAI) is a hybrid modality that combines optical absorption contrast with ultrasound (US) resolution to provide rapid functional and molecular imaging [23]. PAI has previously been used to detect vascular leakage in pre-clinical models, including the brain [24]. However, these injury models often induce excessive tissue damage that does not accurately recapitulate physiological stress (for example, using high-intensity focused US to produce gaps in the blood-brain barrier) [25], however, LPS shares many common inflammatory pathways with surgical trauma and infections [26,27]. Despite challenges related to brain imaging, including skull-induced signal attenuation and acoustic distortion, advances in the production of high-frequency transducers, multispectral analysis, and 3D imaging have improved sensitivity and resolution for transcranial PAI applications in rodents [28]. PAI has been used in *in-vivo* pre-clinical research as early as the 2000s, pioneered by Lihong Wang’s group, where photoacoustic (PA) tomography was used to map hemodynamic changes in cortical blood vessels in the rat brain in response to whisker stimulation [28]. More recently, PA has been used in conjunction with contrast agents such as gold nanoparticles to improve visualization of 0.1 mm vessels in the brain [29], visualize disruption in the blood-brain barrier by passive delivery into brain tissue following focused US [30], and identify intracerebral tumours by accumulation of nanoparticles via the enhanced permeability and retention effect when injected intravenously (IV) [31].

Recent PA neuroimaging has progressed beyond single-wavelength visualization of cerebral vasculature toward volumetric, multi-spectral, molecular, and multimodal measurements. Multi-spectral acquisition can separate oxygenated and deoxygenated hemoglobin contributions and derive parameters such as total hemoglobin and oxygen saturation, while exogenous absorbers extend sensitivity to tracer-associated or molecular processes; however, quantitative interpretation remains affected by wavelength-dependent optical fluence, overlapping absorber spectra, skull-induced attenuation, and the acquisition time required for multi-wavelength imaging [32,33]. Recent developed systems illustrate the value of combining ultrasound and PA measurements. An integrated ultrafast ultrasound and multispectral PA platform enabled concurrent mapping of cerebral blood flow, blood volume, oxygen saturation, and contrast-agent enhancement [34]. Multimodal fluorescence–PAI has also been extended to near-infrared calcium reporters [35], and dual-contrast functional PA microscopy has been used to assess LPS-associated brain lymphatic dysfunction [36]. At a larger spatial scale, co-registered transcranial PA and magnetic-resonance angiography has demonstrated the feasibility of detecting cerebral vascular signals through thinner regions of the adult human skull [37]. Collectively, these developments place contrast-enhanced cerebral PAI within a broader movement toward multiparametric neuroimaging, while emphasizing that single-wavelength signal changes cannot be uniquely assigned to an exogenous tracer when hemoglobin and tracer absorption coexist.

In addition to nanoparticles, molecular dyes such as indocyanine green (ICG) have been used to examine organ perfusion [38], blood-brain barrier disruption [25], and assess cerebral vascular integrity during neurosurgical procedures [39]. ICG provides a strong PA signal due to its high optical absorption, but is prone to photobleaching and has a short biological half life, limiting its use in dynamic or delayed imaging settings [40,41]. To overcome ICG’s limited photostability and biological half-life, ICG can be formulated into ICG J-aggregates (J-aggs), which are highly ordered molecular aggregates first reported over 50 years ago [42]. J-aggs have improved biological stability and photostability compared to molecular ICG, exhibiting a narrow, red-shifted absorption peak (~895 nm) and have enhanced PAI efficiency, making them well-suited for deep-tissue imaging. In-vitro, J-aggs give 2–3 times more PAI signal at J-aggs peak absorption than ICG alone with a matched concentration [43], however, PA signal amplitude can be many orders of magnitude higher *in-vivo* due to increased contributions of spectral colouring/attenuation at lower wavelengths (725 nm, 800 nm) needed to spectrally unmix ICG. Additionally, J-aggs' optical properties are independent of size and concentration, unlike molecular ICG, which shifts its absorption peaks with concentration and overlaps with hemoglobin absorption spectra. Current formulations of J-aggs rely on encapsulation with materials such as liposomes or polymers to reduce polydispersity and maintain stability in biological environments [40]. These modifications introduce complexity in synthesis, increase the risk of immune reaction, and limit reproducibility and regulatory approval. Recently, a novel formulation of azide modified J-aggs substantially improved biologically stability while allowing precise control over size **without encapsulation** [43]. PA signals from J-aggs injected systemically were observed in abdominal organs, including the liver and spleen, demonstrating improved visualization for up to 120 min after injection. J-aggs exhibit a half-life ten times longer than molecular ICG dye and demonstrated improved imaging depth (up to 5 mm from the skin surface).

In this study, we employed a novel formulation of azide-modified J-aggs in combination with a high-energy (50 mJ) laser system and high-frequency transducers capable of deeper tissue imaging (~15 mm). This configuration enabled acquisition of cerebral PA signals in juvenile rats despite skull-related optical attenuation and acoustic distortion. An LPS rat injury model was used to simulate the magnitude of systemic inflammation caused by infections and/or surgical trauma (LPS, 3 mg/kg injected IP) [44], with biologically stable, non-encapsulated J-aggs as PAI tracers.

Introducing contrast agents **systemically** *in-vivo* can enhance PA signal by increasing optical absorption at a specific wavelength at the imaging site. Studies have shown that contrast agent concentration can be altered locally and/or systemically due to the consequences of inflammatory events such as I) extravasation due to leaky, inflamed endothelium (such as lung endothelium) [45]; II) sequestration in organs of the mononuclear phagocyte system including the liver and spleen [46,47]; III) increased phagocytosis resulting from activation of macrophages and microglia [48]. Inflammatory events can also alter contrast agent concentration **locally** by I) restricting blood flow due to infiltration of leukocytes in cerebral capillaries, which could lead to “capillary stall” [49], restricting transport in select cerebral microvasculature; II) hypotension, which is accompanied by vessel dilation [50], which may increase blood volume and consequently the number of contrast agents in some imaging planes [51]. Systemic inflammation can also alter PA signal in the absence of contrast agents by I) decreasing cerebral blood oxygenation [52] and infiltration of leukocytes causing capillary stalls and reducing capillary transport time [49]; II) altered hemodynamics such as reduced cardiac output [53] reducing blood oxygenation systemically; III) hypotension accompanied by blood vessel dilation [50], which can increase PA signal in select imaging planes due to increased blood volume.

To summarize, the consequences of systemic inflammation can alter PA amplitude in the presence and absence of contrast agents at local (organ-specific) and systemic levels by altering contrast agent concentration (locally and/or systemically) and by altering blood oxygenation and blood volume in PAI planes. Assessing the PA signal of cerebral vasculature, which prevents the extravasation of microparticles due to the blood-brain barrier [54], could be used to assess the consequences of systemic inflammation; however, this has not yet been explored. This work uses transcranial contrast-enhanced PAI with J-aggs to monitor local PA signal amplitude and observe indirect consequences of systemic inflammation. This work provides a basis for evaluating contrast-enhanced transcranial PAI as a preclinical method for monitoring inflammation-associated cerebral PA signal changes.

## 2 Methods

### 2.1 Characterization of optical and photoacoustic properties of ICG J-aggregates

J-aggs were synthesized and characterized according to the protocol outlined in our previous work [43]. Briefly, ICG and ICG‑azide stock solutions were mixed in a water-based 20 mM KCl solution at a molar ratio of 10:1 (ICG:ICG-azide) for a total dye concentration of 1 mM. The formulations were incubated for 18 h at 60 °C and purified using three washes at 4000 g for 10 min with Amicon centrifugal filter (100 kDa) to remove the unincorporated dyes. After purification the proper formation of the J-aggs was validated using UV-Vis spectroscopy using a Safire^2^ Microplate Reader (Tecan, Männedorf, Switzerland). Spectrophotometer measurements were performed from 440 nm to 990 nm with a 2 nm step size using a microplate reader containing J-aggs. Concentrations for spectrophotometer measurements were limited to 60 µM due to saturation effects. The hydrodynamic diameter was determined with dynamic light scattering (Zetasizer Nano ZS, Malvern, Worcestershire, United Kingdom). The concentration of dye after purification was estimated using an absorbance standard curve of ICG and J-aggs dissolved using a solution of 10% Triton X-100 after incubation at 37 °C for 10 min. The functionalization with the polyethylene glycol (PEG) groups was performed using DBCO-PEG5k (PG1-DB-5k, Nanocs, New York, USA) incubated with the J-aggs for 3 h at room temperature followed by three washes with Amicon filter (100 kDa) as described above. For functionalization with Cy7, the J-aggs were first modified with DBCO-PEG-biotin followed by conjugation of streptavidin modified with Cy7 (AAT Bioquest, Pleasanton, USA) and purified using Amicon filter. The PA signal was recorded using a PAI system (Vevo F2 LAZR-X, VisualSonics, Toronto, Canada) equipped with a tunable nanosecond pulsed laser (680–970 nm) and UHF29X (linear array transducer with 256 elements, central frequency 21 MHz, −6 dB bandwidth: 15–29 MHz). PA measurements were performed between 680 nm and 970 nm with a 1 nm step size, using J-aggs contained in polyurethane phantom tubing (inner diameter = 0.381 mm, outer diameter = 0.832 mm, Scientific Commodities Inc., Lake Havasu City, USA). Polyurethane tubes were constrained to a phantom holder and submerged in Mili-Q water. Concentrations for PA measurements ranged from 0.8 µM to 100 µM diluted in 0.9% normal saline (Jolly’s Medical, Regina, Canada). A cross section of the tubes was imaged with 10 frames taken and averaged at each wavelength. PA signals were averaged over 5 spatial locations spaced 5 mm apart in the tubing.

### 2.2 Rat Model of LPS-induced systemic inflammation

Animal experiments were approved under ACC Protocol #220921 at St-Michael’s Hospital (part of Unity Health Toronto). Rats were socially housed, allowed free access to water and chow with 12-hour light-dark cycles. Three-week-old female Sprague Dawley rats (Charles River Canada, Senneville, QC) were used because of the thin cranial bones allowing for sufficient optical and acoustic transmission. Rat’s weights ranging from 40 to 70 g and were randomly assigned to experimental groups receiving a single IP injection (~75 µL) of 3 mg/kg LPS (*Escherichia coli* O111:B4, Sigma-Aldrich, Oakville, Canada; n = 6) or 0.9% normal saline sham (n = 5). The study is a pilot performed for effect-size estimation; thus, a power calculation was not done prior to imaging. However, the PAI data was used to estimate power for future studies and is included in the Supplemental Figure 6. Females were used in this study to reduce variance from sex-dependent LPS dose response, inflammatory response due to sex differences will be investigated in future studies. Rats were anesthetized with 2.5% isoflurane (Baxter, Illinois, USA) and 100% medical oxygen at 1 L/min during imaging. Supplemental heat was administered using a homeothermic control module (RightTemp, Kent Scientific, Torrington, USA) to maintain core temperature at 37°C. A custom 3D-printed stereotactic holder was used to ensure consistent skull positioning between imaging time points. Before imaging, the scalp was shaved, and the remaining hair was removed with depilatory cream (Nair, Church & Dwight Co Inc., Ewing, New Jersey, USA), applied for 30 s and removed with cotton gauze. Following PA imaging 4-hours after IP injection, rats were overdosed with 5% isoflurane for 5 min, and a thoracotomy was performed to gain access to the heart for a cardiac puncture. Blood draws occurred approximately 5.5 h after IP injection for each animal. 1 mL of blood was collected in 10% citrate and centrifuged at 1000*g* for 10 min to isolate plasma. 200 µL of plasma was collected by pipette for each animal after centrifugation and stored at −80°C until it arrived at Eve Technologies for analysis.

### 2.3 B-mode and PA-mode image acquisition and image processing

PAI and co-registered B-mode were performed using the Vevo F2 LAZR-X with a UHF29X transducer. The imaging setup is shown in Fig. 1. Rats were positioned supine on a custom-built platform consisting of a galvanized steel plate with a 5 cm x 10 cm central imaging window. The transducer was placed beneath the imaging window and coupled directly to the scalp with US gel. The transducer was mounted on a motorized stage to facilitate 3D imaging. Baseline PAI was performed before the IP injection. Approximately 5 min after IP administration of LPS or saline, animals received the first IV bolus of J-aggs (~0.93 mg/kg – 150 µL at 400 µM) and PA imaging was initiated approximately 5 min later. At 2 h after IP treatment, PA imaging was performed before the second J-aggs dose; the second IV bolus was then administered at approximately 2 h 5 min, followed by PA imaging approximately 5 min later. Final PA imaging was performed 4 h after IP treatment, approximately 1 h 55 min after the second J-aggs injection. The second IV dose of J-aggs was administered to restore circulating contrast before the later imaging interval and to evaluate the subsequent cerebral PA response after additional progression of inflammation in the LPS model. 2-hours was chosen based on previous work, which showed residual PA signal from J-aggs up to 120 min following injection [43].

**Fig. 1:**
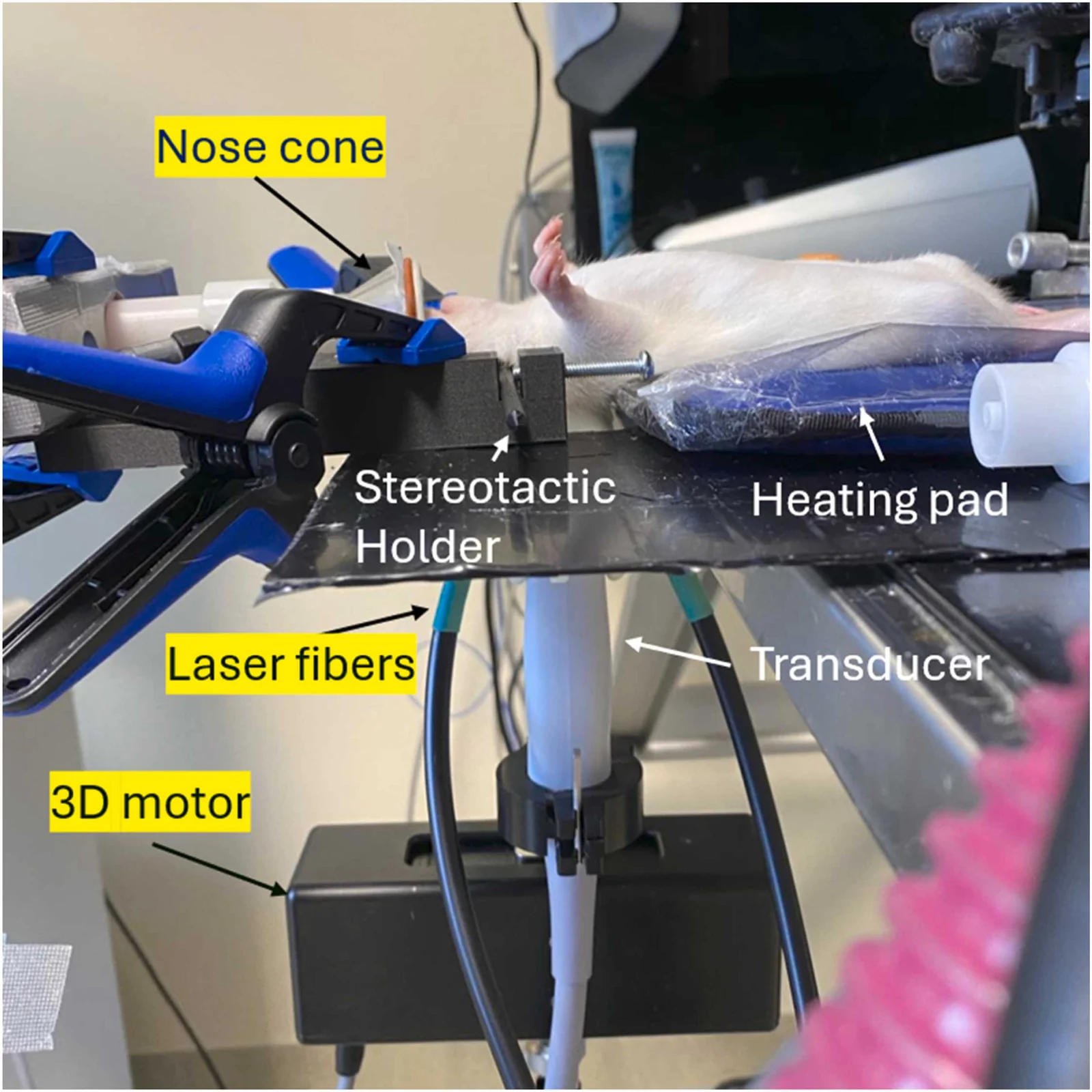
Photoacoustic imaging setup in a custom platform using the Vevo F2 LAZR-X. 3-week-old female Sprague-Dawley rats were placed atop a steel plate with their heads positioned above an imaging window. A 3D-printed stereotactic holder is used to maintain positions between imaging time points. An electric homeothermic heating pad is used to maintain and monitor physiological temperature. The transducer is attached to a 3D motor and positioned beneath the steel plate and coupled with ultrasound gel directly to the rat’s scalp.

*In-vivo* 3D PA data were acquired using the Vevo F2 LAZR-X system with the UHF29X linear-array transducer in RF mode at an optical wavelength of 890 nm and a PA gain of −9 dB. No frame averaging was applied to the *in-vivo* PA acquisitions. Each 3D dataset comprised 40 imaging planes spaced 0.15 mm apart, and pulses were delivered at the system’s default repetition frequency of 20 Hz. Single-wavelength 3D acquisition was used because stable 3D multi-wavelength acquisition was not technically feasible with the software version available during this study. Specifically, attempts to initiate multi-wavelength 3D sweeps resulted in system-level acquisition errors requiring restart. Although sequential single-wavelength 3D scans at multiple wavelengths could theoretically have been acquired, this approach would have introduced substantial risk of respiratory motion, transducer–animal repositioning, coupling changes, inconsistent timing between wavelengths, and scan-to-scan variation during post-processing. These artifacts would have made spectral unmixing unreliable for the present longitudinal study. Sequentially translating a single-plane multispectral acquisition across all 40 imaging planes was also considered but would have substantially prolonged acquisition, increasing the likelihood of contrast-agent washout and time-dependent spectral changes during the scan. Consequently, the present analysis was restricted to single-wavelength 890 nm imaging, and the reported P90 values were interpreted as composite PA signal changes rather than spectrally unmixed J-aggs-specific measurements. Because a large portion of the imaging planes contained reverberation artifacts from the skull, 11 imaging planes were used, centred on the subependymal arteries (an area with significant PA SNR at depth), as shown in Fig. 3 and Fig. 4. Comparisons were made between LPS-treated and sham animals to evaluate inflammation-associated differences in composite cerebral PA signal following J-aggs administration. PAI was conducted at five timepoints (Fig. 2) corresponding to the following: (I) baseline imaging, before injection of saline/LPS (t = -5 min) (II) after the first IV injection of J-aggs (t = 10 min) (III) 2-hours after injection of saline/LPS (t = 2-hours), (IV) after the 2nd dose IV J-aggs (t = 2-hours, 10 min) (v) 4-hours after saline/LPS (t = 4-hours). The imaging timeline was designed to capture dynamic changes of PAI enhancement due to J-aggs presence and clearance. To reduce longitudinal repositioning error, animals were placed in a custom stereotactic holder at each imaging session, and the 3D acquisition volume was positioned using B-mode anatomical landmarks, including the subependymal and lenticulostriate arteries. Eleven consecutive imaging planes centred on these landmarks were selected at each timepoint for analysis. No fiducial markers or rigid or deformable image-registration algorithms were used; therefore, residual differences in probe angle, coupling, and position cannot be excluded. For each animal and imaging timepoint, a manual freehand region of interest (ROI) was drawn on each of the 11 selected B-mode coronal planes centred on the subependymal arteries. The ROI encompassed all visible brain parenchyma within the echogenic inner skull–brain interface and excluded the skull, scalp, ears, extracranial muscle, and acoustic-shadow regions outside the brain. Where the parenchymal boundary was obscured by acoustic shadowing or reduced B-mode contrast, the contour was interpolated smoothly between adjacent visible boundary segments according to the expected rounded geometry of the brain cross-section. PA signal intensity was not used to define the ROI boundary. Each B-mode contour (Fig. 3) was then transferred unchanged to the corresponding co-registered PA image, (Fig. 4) and the 11 plane-specific ROIs were combined into a 3D cerebral ROI for P90 analysis.

**Fig. 2:**
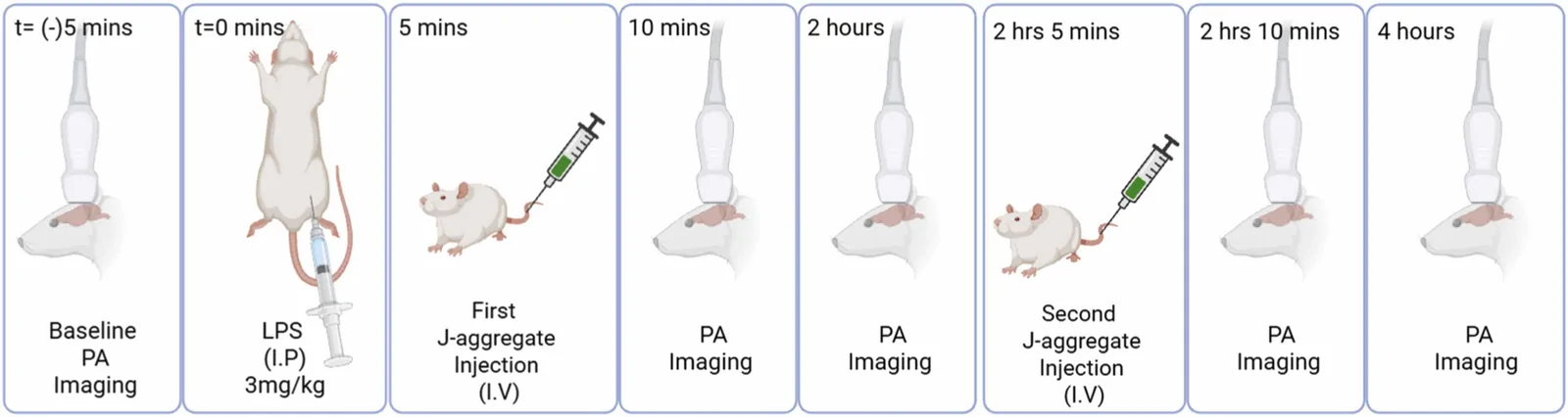
Experimental timeline. Baseline imaging was performed before intraperitoneal (IP) administration of LPS or saline and before intravenous indocyanine green J-aggregate (J-aggs) administration. The first J-aggs dose was administered approximately 5 min after the IP treatment, followed by PA imaging approximately 5 min later. The second J-aggs dose was administered approximately 2 h 5 min after IP treatment, followed by PA imaging approximately 5 min later. Final PA imaging was performed at 4 h after IP treatment, approximately 1 h 55 min after the second J-aggs injection. [Created with BioRender].

**Fig. 3:**
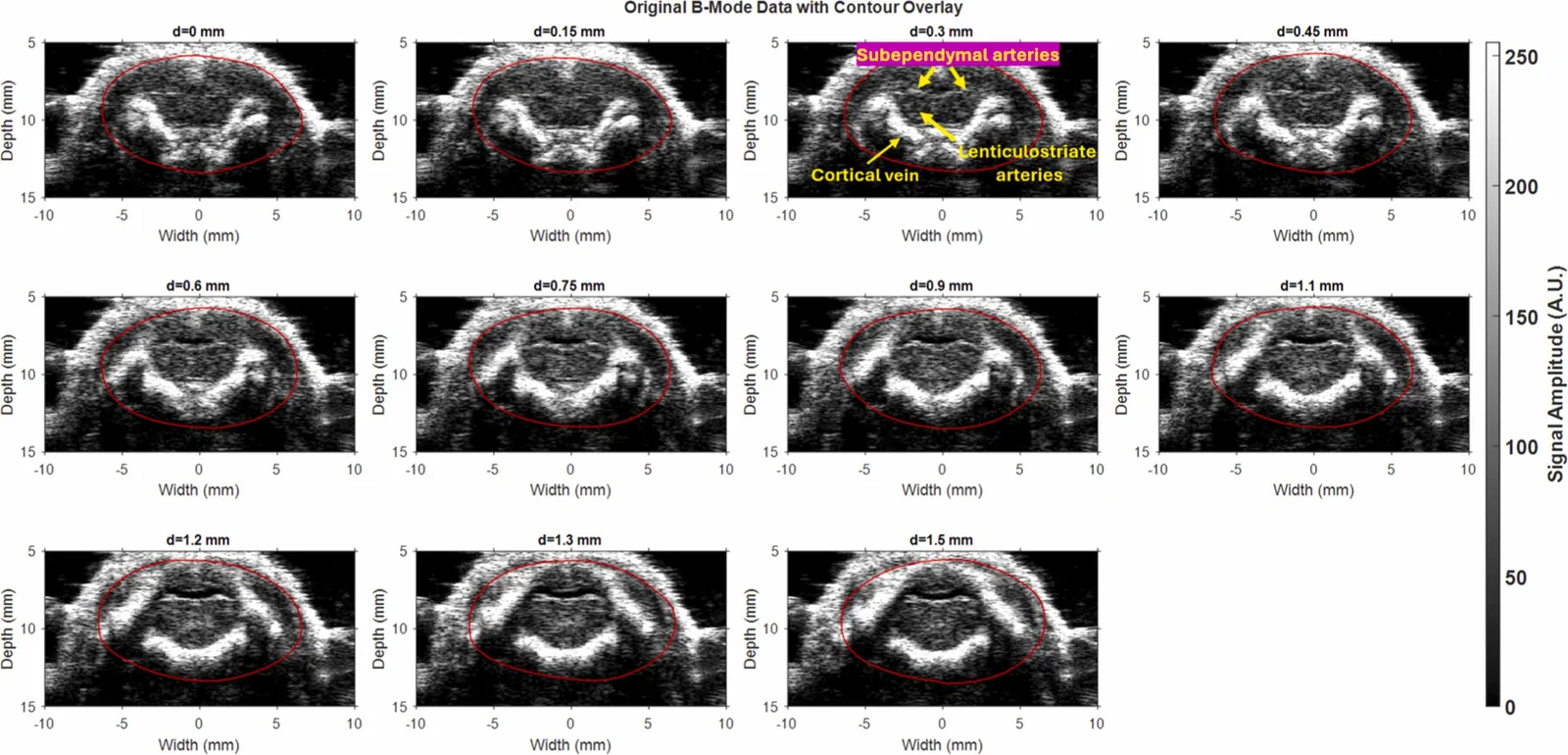
Representative ultrasound B-mode coronal sections of the rat brain where several anatomical landmarks can be identified, including: 1) cortical vein; 2) subependymal arteries; and 3) lenticulostriate arteries. Contours used for analysis are shown in red. Contours were drawn to include all brain parenchyma, excluding cranial bones and ears, shown by a delineation in the B-mode. Images were taken before lipopolysaccharide and indocyanine green J-aggregates injections (baseline, t = -5 min). “d” represents the distance travelled by the transducer from the base of the skull near the spinal cord towards the front of the head. The color bar indicates the B-mode signal amplitude in arbitrary units.

**Fig. 4:**
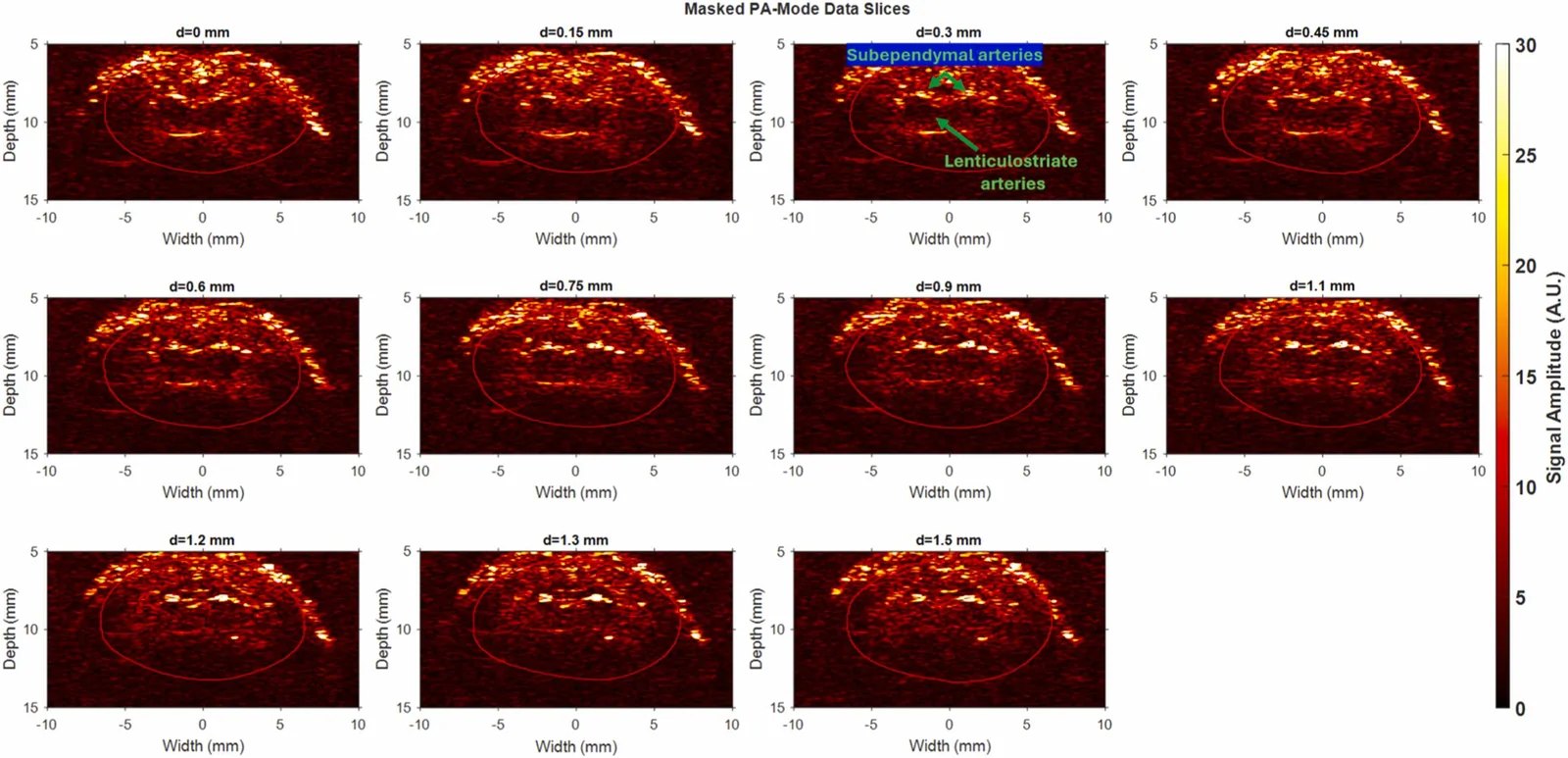
Representative photoacoustic (PA)-mode coronal sections of the rat brain where the subependymal arteries and lenticulostriate arteries are identified. Contours from ultrasound B-mode images (shown in red) were transposed onto co-registered PA-mode images. Images were taken before lipopolysaccharide and indocyanine green J-aggregates injections (baseline, t = -5 min). “d” represents the distance travelled by the transducer from the base of the skull near the spinal cord towards the front of the head. The color bar indicates the PA signal amplitude in arbitrary units.

The LAZR-X integrated illumination delivers nominal pulse energies of up to 50 mJ at the output of the dual-arm fibre bundle, distributed across two ~14 × 1 mm strip-illumination apertures positioned on either side of the UHF29X transducer. Assuming negligible coupling and absorption losses through the ultrasound gel layer, the per-pulse fluence at the scalp surface is approximately 178 mJ/cm² per arm. Pulses were delivered at the system's default pulse repetition frequency of 20 Hz. Per-pulse energy was not measured directly at the scalp, and depilated skin is more reflective and less attenuating than intact furred skin; therefore, the surface fluence reported here is an upper-bound estimate, and the in-tissue fluence reaching the cortex is substantially lower due to attenuation by skin, skull, and overlying tissue. Direct fluence measurement and Monte Carlo modelling of in-tissue light transport will be the subject of future work.

To account for pulse-to-pulse variability, PA images were normalized based on individual optical pulse energies. For each image acquisition, energy readings were averaged across four image quadrants and raw pixel image data were divided by the mean energy to yield energy-compensated images. All image processing was done using custom-written MATLAB code. PA signal within the cerebral ROI is heterogeneous, with high-amplitude voxels arising from contrast-enhanced macrovasculature (e.g., subependymal arteries) and low-amplitude voxels from microvasculature, parenchyma, and a noise floor comprising electronic, thermal, and optically translucent contributions. A whole-ROI mean is dominated by the low-amplitude majority and dilutes signal change in the contrast-bearing population. We therefore thresholded each image at the 90th percentile of voxel intensity within the ROI and report the mean of voxels above this threshold (P90), which preserves sensitivity to changes in the high-amplitude vascular PA signal while remaining operator-independent. The P90 metric, corresponding to the mean of the upper 10% of voxel intensities, was pre-specified based on prior measurements and used as the primary endpoint. A threshold sensitivity analysis using a linear mixed effects model structure is shown in Supplemental Figure 3. The baseline-4-hour group × timepoint contrast remained negative across tested top-pixel thresholds, ranging from −46.3% to −35.7%, with subject-level bootstrap 95% CIs excluding zero at all thresholds. Baseline-2-hour and 2-hour-4-hour contrasts had bootstrap 95% CIs crossing zero across all thresholds. (Supplemental Figure 3) Representative histograms for pixel intensities can be found in Fig. 7.

In addition to 3D single wavelength acquisitions, 2D single-plane multispectral PA acquisitions (680–970 nm) were additionally collected in the same coronal plane where the subependymal and lenticulostriate arteries were located, to explore whether spectral information could help distinguish J-aggs–specific signal from endogenous hemoglobin absorption. These scans were acquired prior to the primary 3D single-wavelength (890 nm) dataset used for the P90 analysis described above. However, probe position was not fixed across sessions using fiducial markers and thus a high degree of anatomical drift was observed during longitudinal imaging, which could lead to significant variability in data unrelated to J-aggs concentration or consequences of inflammation. Energy-normalized multispectral PA spectra and the corresponding B-mode images used to generate the whole-brain ROI at each timepoint are provided in the supplemental material (Supplemental Figures 4–5).

### 2.4 Histological assessment of cerebral inflammation

To perform a histological assessment, thoracotomies were performed following an anesthetic overdose (5% isoflurane mixed with 100% oxygen, administered for 5 min), and the right atrium was cut to allow blood to flow freely out of the heart. 20 mL of Phosphate Buffered Saline (PBS) was injected into the left ventricle with a 25 G needle, followed by 20 mL of 4% PFA (Sigma Aldrich, Oakville, Canada). Brains were isolated using curved scissors, stored in 4% PFA for 12-hours, then washed with PBS and transferred to 30% sucrose for 72-hours. Samples were washed again with PBS and then frozen in optical cutting temperature compound (OCT, Sakura Finetek, Torrance, USA), stored at −80°C. Brain tissues were sectioned coronally in a cryostat at a thickness of 20 microns, with tissues harvested every 0.5 cm from the front of the brain to the spinal cord.

Histological examination of brain tissues was conducted as previously reported [55]. In brief, tissues were incubated in horse blocking buffer (composed of 10% horse serum, H1270, Sigma Aldrich, Oakville, Canada) for 1 h at room temperature to permeabilize the tissues. Primary and secondary antibodies were diluted in an antibody buffer according to the manufacturer's specification (anti-laminin – 1:1000, anti-VCAM-1 – 1:250) and secondary antibodies were diluted 1:400. Anti-laminin polyclonal antibodies and secondary antibody Alexa Fluor 594 (AB_2340858, Jackson ImmunoResearch Laboratories, West Grove, USA) were used to visualize laminin in the basement membrane of brain vasculature (shown in purple in Fig. 10). Anti-VCAM-1 monoclonal antibody (EPR5047, Abcam, Waltham, USA) and secondary antibodies Alex Fluor 647 (AB_2340625, Jackson ImmunoResearch Laboratories, West Grove, USA) were used to visualize expression of VCAM-1 (shown in blue, Fig. 10), a surface marker that is overexpressed on activated endothelial cells and a key indicator of local inflammation. In a subset of experiments, J-aggs were covalently bonded with Cy7, a fluorescent dye, to allow for confirmation/visualization of J-aggs in cerebral vasculature (shown in green, Fig. 11). Slides with primary antibodies were incubated overnight at 4 °C in a humidified chamber, then washed 3 times in a 0.05% Tween-PBS (Tween 20, Sigma-Aldrich, Oakville, Canada; PBS 1X, Wisent Inc., Saint-Jean-Baptiste, Canada) solution for 5 min. Slides were then incubated with secondary antibodies for 2-hours at room temperature, then washed 3 times in 0.05% Tween-PBS for 5 min and mounted with Fluoroshield. (Sigma-Aldrich, Oakville, Canada) Slides were imaged using an AxioImager 2 (Zeiss, Jena, Germany) at 20x magnification, and images were tiled to allow a full field of view of the tissue sections. Image analysis was performed using ZEN Pro software. Fluorescence intensity was summarized descriptively across three tissue sections from one animal in each treatment group. Because these sections were obtained from the same animal, they were treated as within-animal technical observations rather than independent biological replicates; therefore, no inferential statistical comparison of VCAM-1 fluorescence between treatment groups was performed.

### 2.5 Cytokine panel to confirm LPS-induced systemic inflammation

The cytokines that were assessed include INF-γ, IL-1α, IL-1β, IL-2, IL-4, IL-6, IL-10, IL-17A, Leptin, MCP-1, MIP-1α, RANTES, and TNFα. These are classic systemic markers of inflammation and were assessed to ensure LPS injections elicited systemic inflammation [56,57]. The cytokines were assessed in duplicate with a multiplex method (rat cytokine 27-Plex Discovery Assay, Eve Technologies, Calgary, Canada).

### 2.6 Statistical analysis

P90 was calculated at each imaging timepoint, and each animal was normalized to its own pre-injection baseline according to ΔP90(%) = (P90ₜ − P90baseline) / P90baseline × 100, where P90ₜ is the absolute P90 at timepoint t. All subsequent references to normalized values denote ΔP90(%), whereas P90 denotes the absolute quantity. Accordingly, baseline ΔP90(%) values were defined as 0%, and positive or negative values represent increases or decreases relative to that animal’s baseline. Statistical analysis was performed in JMP Pro (SAS, North Carolina, USA). A linear mixed-effects model was used to assess statistical significance across fixed effects of treatment group×time, with animal identification number categorized as a random effect. Timepoints were restricted to baseline (before J-aggs IV injection), 2-hours (before the 2nd J-aggs IV injection), and 4-hours. PA imaging immediately following the J-aggs injections was not included in statistical analysis as transient increases in PA signal immediately following injection may not provide an accurate representation of LPS treatment effects. Instead, analysis was structured to compare the baseline PA signal to PA signal 2-hours and 4-hours after LPS treatment. The purpose of the PA imaging taking place immediately after J-aggs injections was to confirm injections were performed successfully. The Mixed Effects Model did not show a significant difference for group×time between baseline-2-hours after IP saline/LPS, and 2-hours-4-hours after IP saline/LPS, however, showed significance for baseline-4-hours (p = 0.0262) after IP saline/LPS. Because three group×time contrasts were evaluated (baseline–2-hours, 2-hours–4-hours, and baseline–4-hours), the p-values reported for the PA endpoint are unadjusted for multiplicity; the baseline–4-hours contrast would not meet a Bonferroni-corrected threshold across three contrasts (α=0.0167). The cytokine panel, by contrast, was corrected using the Benjamini–Hochberg procedure. This difference in adjustment between the two outcome families is noted so that the PA result is interpreted as an exploratory, hypothesis-generating finding. To assess robustness to this heavier-tailed residual behaviour, the linear mixed effects model was refit using subject-level bootstrap resampling; for the primary top 10% pixel metric, the baseline-4-hour group × timepoint contrast remained negative, with an estimate of −41.7% and a bootstrap 95% CI of −68.5% to −16.1%. (Supplemental Figure 2) Model fit was summarized using measured-versus-conditional-predicted values, Bland-Altman analysis, and leave-one-animal-out error estimates. (Supplemental Figure 1)

A prospective power analysis was performed to support planning of future vehicle-control experiments. The analysis estimated the number of animals required per group to achieve 80% power across assumed between-group differences in cerebral PA signal and the power achievable with n = 6 animals per group (Supplemental Figure 6). An assumed 5% PA-signal difference was included as an illustrative small contrast-independent effect. This scenario was informed by a previous neonatal rat study reporting an approximately 10% reduction in regional cerebral blood-oxygenation following LPS-induced inflammatory injury [58]. The 5% PA-signal difference was used as a modelling assumption rather than a direct conversion of oxygen saturation to PA amplitude and was not estimated from the present dataset.

## 3 Results

### 3.1 Absorbance and photoacoustic signal from indocyanine green J-aggregates *in-vitro*

Using our previously published protocol, we synthesized J-aggs with a diameter of ~903 nm (± 87 nm) for a polydispersity index of ~0.4 (± 0.06) [43]. Results for J-aggs optical absorbance and PA signal are shown in Fig. 5. For PA measurements, J-aggs were injected into polyurethane medical tubing (inner diameter = 0.381 mm, outer diameter = 0.832 mm, Scientific Commodities Inc., Lake Havasu City, USA) and submerged in Mili-Q water. While peak absorption in the PA measurement is accurate within 5 nm of the spectrophotometer measurements (890 nm in PA vs. 895 nm in spectrophotometer), the peak in the spectrophotometer is much narrower. PA measurements also show much higher absorption in shorter wavelengths (700–885 nm) compared to spectrophotometer measurements. This may be due to high energy pulses used in PA which could lead to transient degradation of J-aggs into ICG monomers and dimers. More information regarding J-aggs optical properties, size distribution, and chemical structure can be found in previously published work [43].

**Fig. 5:**
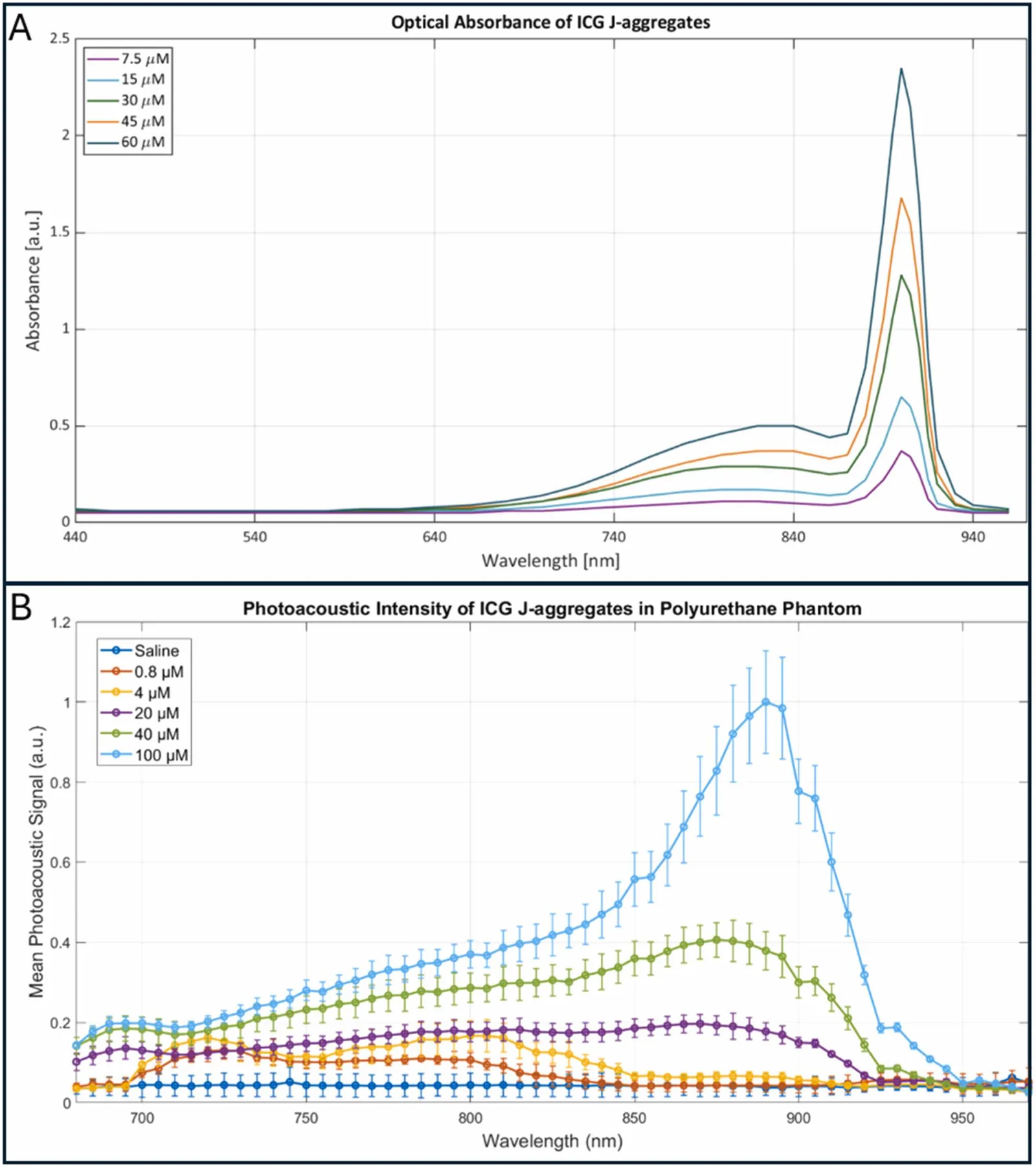
Optical and photoacoustic properties of indocyanine green J-aggregates (J-aggs). (A) UV–Vis–NIR absorbance spectra showing a peak near 895 nm. (B) Normalized photoacoustic spectra showing a peak near 890 nm and broader signal at shorter wavelengths at lower concentrations. Data were averaged across five spatial locations; error bars represent one standard deviation. Saline-filled tubing is shown for reference.

### 3.2 Lipopolysaccharide group exhibited decreased average P90 compared to sham

At 890 nm, PA signal within the cerebral ROI reflects a composite contribution from J-aggs and endogenous absorbers, primarily hemoglobin. Therefore, the P90 values reported here were interpreted as changes in composite cerebral PA signal rather than direct measurements of J-aggs concentration or biodistribution. Representative PA images from each group are shown in Fig. 6. Baseline PA images between sham and LPS groups were compared using a Student’s *t*-test and were not significantly different (p = 0.0659), although baseline variability may reflect differences in endogenous hemoglobin-related signal. Higher ΔP90(%) values were observed in the sham group at 2 and 4 h after IP injection compared with the LPS group, with increased contrast in deeper vascular structures such as the subependymal arteries. (Fig. 6)

**Fig. 6:**
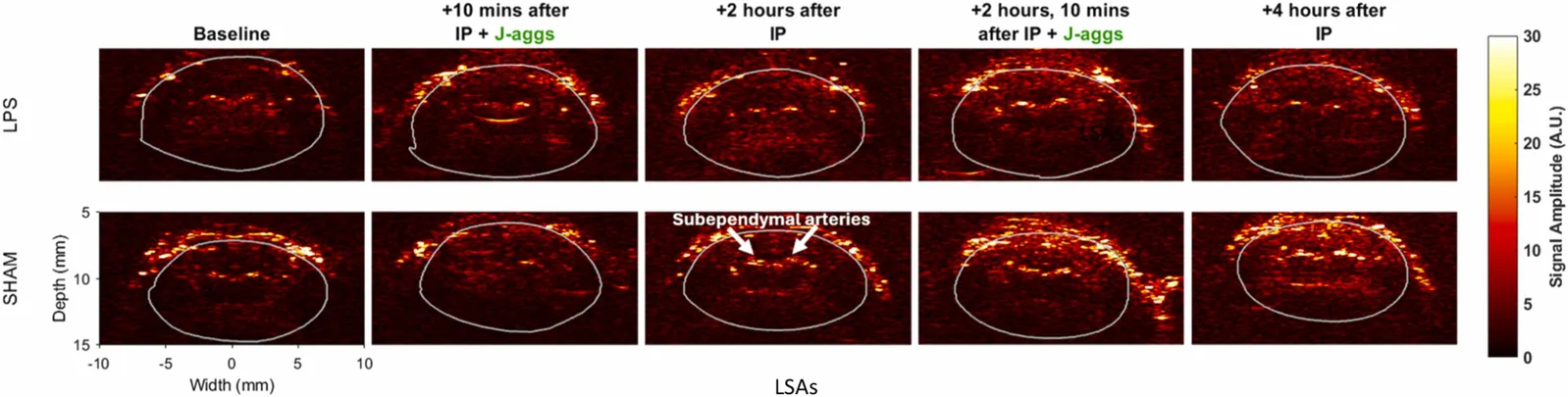
Representative photoacoustic (PA) image data taken from 1 centralized spatial location in each timepoint from a lipopolysaccharide (LPS)-treated animal (top) and a sham animal (bottom). Timepoints are shown for baseline (before IP/IV injections), 10 min after IP LPS/saline + IV J-aggs, 2-hours after IP LPS/saline, 2-hours, 10 min after IP LPS/saline + J-aggs, and 4-hours after IP LPS/saline. Increased PA signal is observed for deeper tissues, including the subependymal arteries of the brain indicated with the white arrow after IV J-aggs injection.

Histograms from the contoured PA data were generated to visualize the distribution of pixel intensities within the ROIs (Fig. 7). Contoured ROI data was used to generate a 3D array in MATLAB. A threshold was used to exclude the bottom 90% of pixels in the 3D array and the P90 was calculated (rather than the overall mean) to reduce affects of heterogeneity in images and contributions from low signal intensity which may be composed of electronic/thermal noise and optically translucent tissue. The total number of pixels in the contoured images and resulting number after exclusion is shown. Histogram statistics including the minimum, maximum, mean, and median prior to thresholding can be found on the bottom right of the images. A sensitivity analysis was conducted by recalculating the PA outcome as the mean signal intensity from the top 5%, 10%, 15%, 20%, and 25% of voxel intensities within the cerebral ROI, corresponding to percentile thresholds from the 95th to 75th percentile. The primary P90 metric corresponded to the mean of the upper 10% of voxel intensities. The baseline-4-hour group × timepoint interaction remained negative across the tested thresholds and was strongest at higher percentile thresholds, whereas the baseline-2-hour and 2-hour-4-hour contrasts were not consistently supported. Detailed p-values, contrast estimates, and bootstrap confidence intervals are provided in Supplemental Figure 3.

**Fig. 7:**
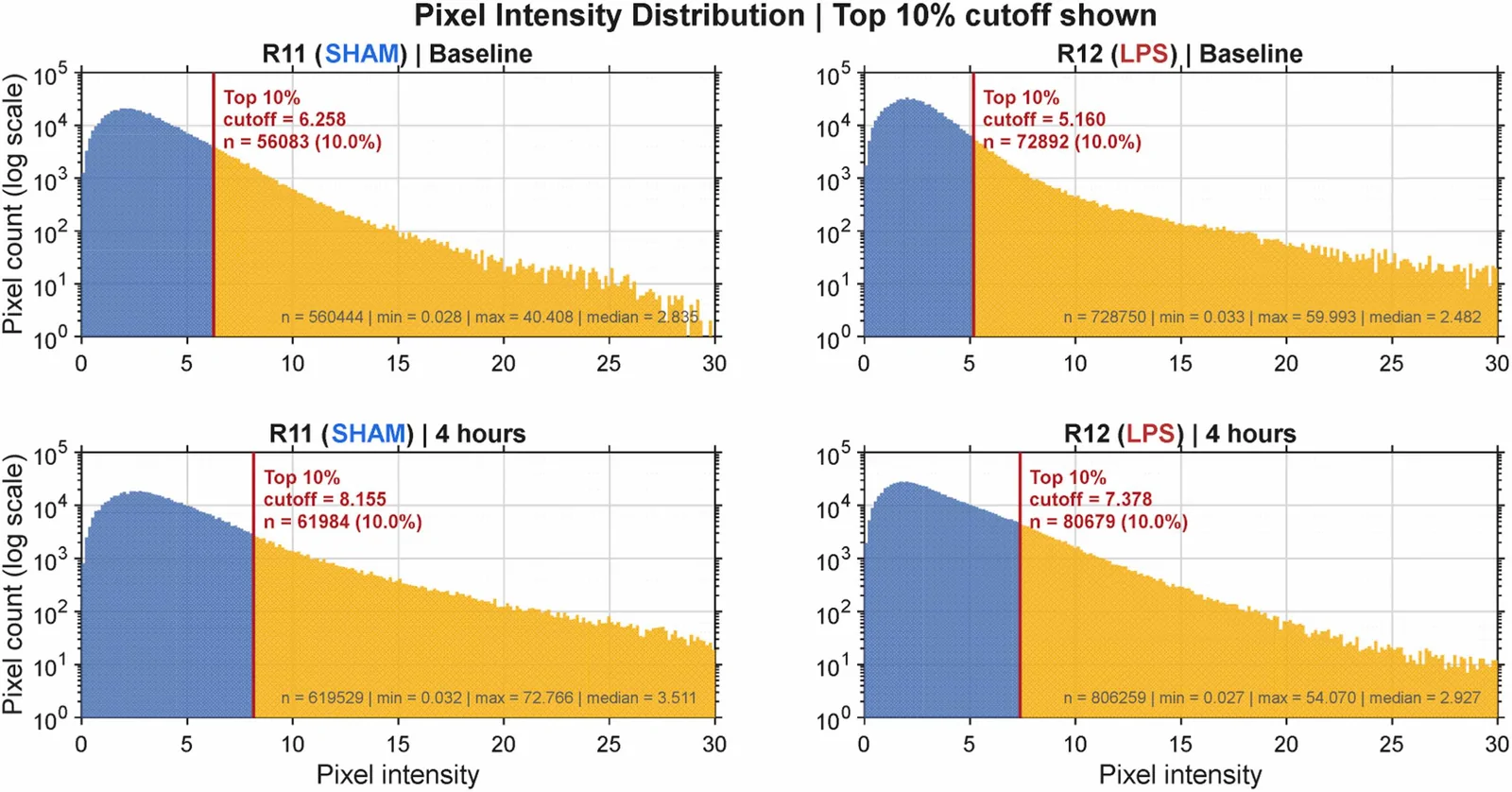
Representative cerebral photoacoustic pixel-intensity distributions from one sham-treated rat and one lipopolysaccharide-treated rat at baseline and 4 h. The 90th-percentile threshold separates the upper 10% of voxel intensities used to calculate P90 from the remaining voxels. Threshold values and descriptive distribution statistics are displayed within each panel.

Fig. 8 shows a box-and-whisker plot of ΔP90(%), defined as the percent change in P90 relative to each animal’s baseline PA image; IV J-aggs injections are indicated on the figure. Imaging performed after J-aggs IV injection show an increase in PA signal for both groups from the baseline suggesting a successful injection. PA signal is not significantly different 2-hours after IV J-aggs injection, which can indicate extended circulation half-life of J-aggs. An increase in PA signal is observed after the 2nd IV injection of J-aggs, and a separation between sham and LPS groups can be observed between baseline and 4-hours after IP saline/LPS which was found to be significant when group × timepoint was analyzed in a Linear Mixed Effects Model (p = 0.0262). Power was not computed retrospectively for the observed contrasts, since power calculated from the realized effect size is a deterministic restatement of the p-value and does not provide independent evidence about the reliability of the result. Instead, the observed effect sizes were used prospectively to inform the design of future experiments: assuming an effect size comparable to that observed here, approximately 14 rats per group would be required to detect a group difference at the 2-hour timepoint with 80% power. Because these measurements were acquired at a single wavelength, the observed divergence between sham and LPS groups cannot be attributed exclusively to J-aggs biodistribution. Potential contributors include altered J-aggs circulation or cerebral delivery, as well as LPS-induced changes in cerebral blood volume, oxygenation, perfusion, and hemoglobin absorption at 890 nm.

**Fig. 8:**
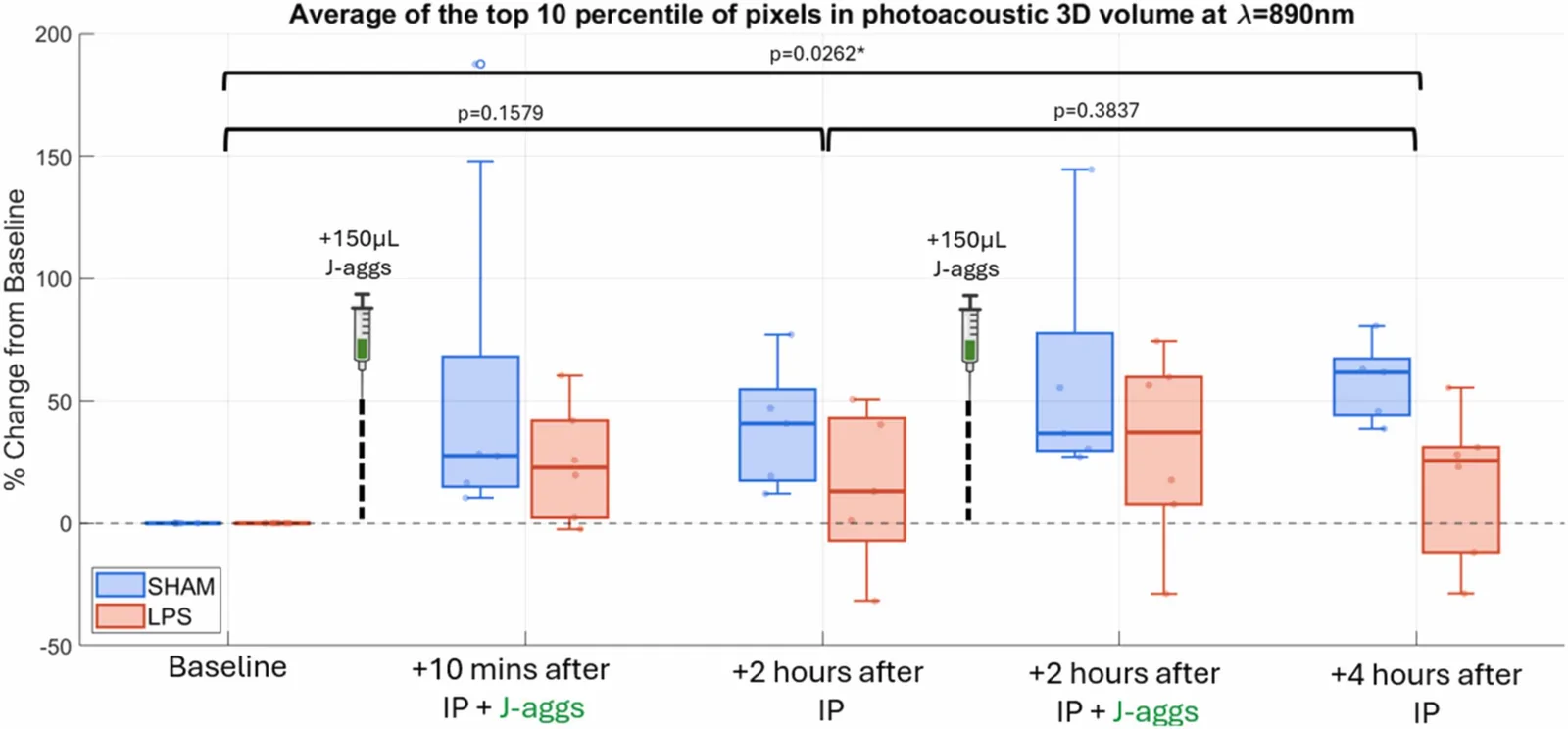
ΔP90(%), the change in cerebral P90 relative to baseline, in sham-treated rats (n = 5) and LPS-treated rats (n = 6). P90 represents the mean intensity of the upper 10% of pixels within the cerebral region of interest and, at 890 nm, reflects composite signal from J-aggs and endogenous absorbers. J-aggs injection times are indicated. Group × time comparisons were evaluated using a Linear Mixed Effects Model; the baseline-4-hour interaction was significant (p = 0.0262).

Exploratory 2D single-plane multispectral PA acquisitions (680–970 nm) were also collected at each timepoint to assess whether spectral shape could help resolve J-aggs–specific contributions from the composite 890 nm signal (Supplemental Figures 4–5). Qualitatively, the spectra acquired approximately 5 min after IV J-aggs administration retained a broad longer-wavelength feature in the approximate 880–910 nm range and did not show an obvious isolated relative increase centered in either the approximate 715–725 nm ICG dimer region or the 780–800 nm ICG monomer region. This observation is consistent with persistence of a J-agg-associated spectral component after injection but does not exclude partial conversion to monomeric or dimeric ICG because fluence correction, spectral unmixing, and anatomically stable 3D multispectral acquisition were not performed (Supplemental Figure 5). However, comparison of the B-mode images used to generate the whole-brain ROI across timepoints showed that the same coronal plane and anatomical landmarks were not consistently reacquired within a given animal, with visible shifts in probe position and slice depth over the imaging session (Supplemental Figure 4). Because the ROI did not correspond to an identical brain region at each timepoint, differences in energy-normalized spectra between groups and across time are not presented as evidence of J-aggs-related spectral change and are reported in the supplemental material without quantitative spectral unmixing or group-level interpretation.

### 3.3 Cytokine panel confirms increased levels of cytokines following lipopolysaccharide injection compared to sham group

To assess the inflammatory responses plasma cytokine levels were compared between sham and 3 mg/kg IP LPS groups. The results of the cytokine panel are shown in Fig. 9. Pro-inflammatory cytokines IL-1β,IL-1α, IL-17A, and TNFα were all significantly elevated in LPS animals compared to sham. Similarly, chemokines MCP-1, MIP-1α, and RANTES were increased. Anti-inflammatory cytokine IL-10 and leptin, a pleiotropic hormone with known pro-inflammatory properties was also found to be significantly increased in the LPS group. A two tailed unpooled *t*-test showed nine of the tested cytokines, chemokines, and hormones were statistically significant (p < 0.05) between sham and LPS group. A post-hoc Benjamini-Hochberg procedure with a false discovery rate of 0.05 was performed and all nine cytokines were deemed significant.

**Fig. 9:**
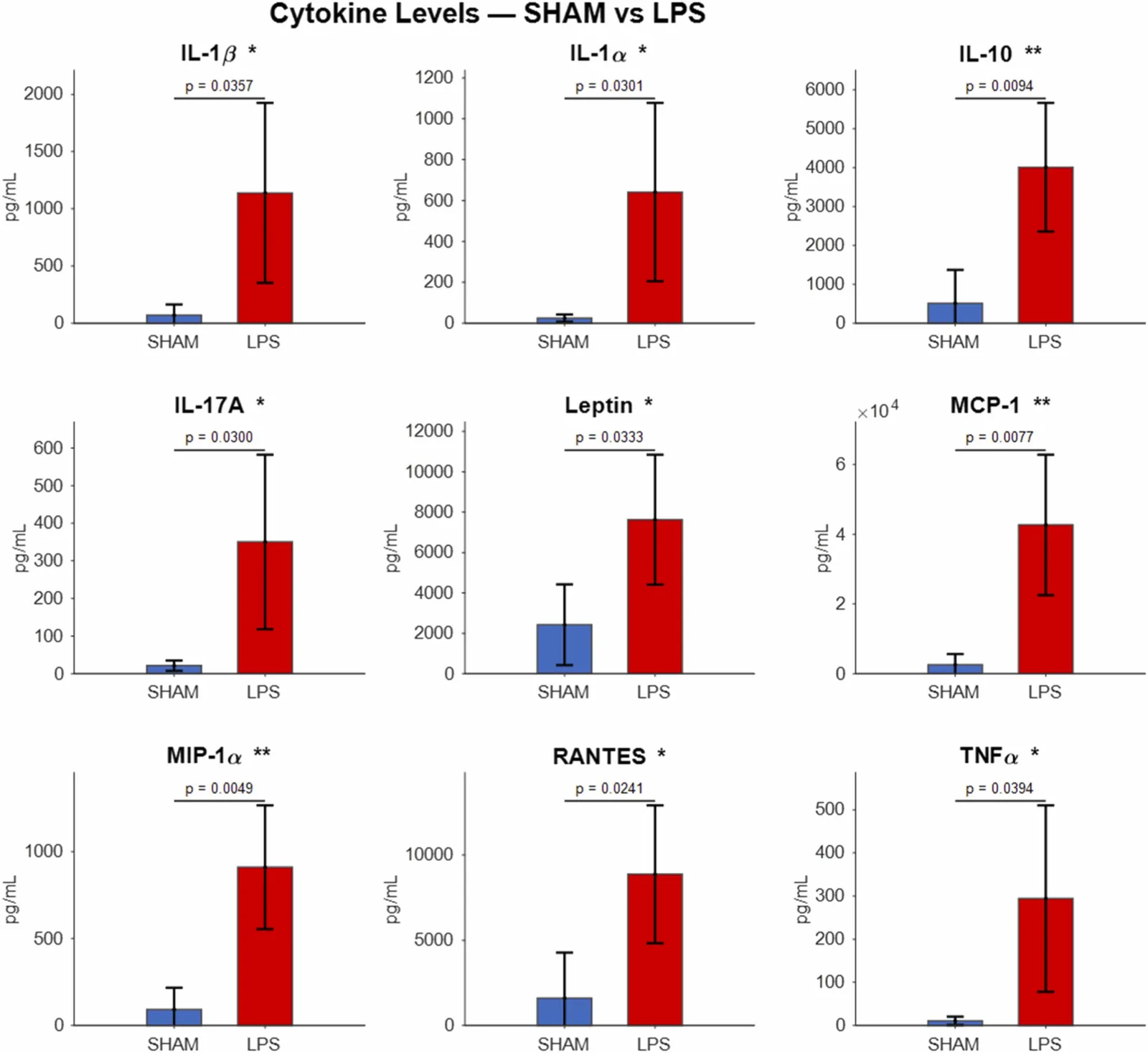
Plasma inflammatory-marker concentrations in sham- and LPS-treated rats. Data are shown as mean ± standard deviation (n = 4 per group). Between-group differences were evaluated using two-tailed unequal-variance *t*-tests followed by Benjamini–Hochberg correction. *p < 0.05; **p < 0.01.

### 3.4 Representative immunohistochemistry shows presence of VCAM-1 in brain parenchyma and J-aggs in cerebral vasculature five hours after lipopolysaccharide injection

Representative VCAM-1 immunofluorescence from one sham-treated and one LPS-treated animal is shown in Fig. 10. Greater VCAM-1-associated fluorescence was observed in the LPS-treated specimen across the three tissue sections examined from each animal. However, because tissue loss during freezing and sectioning limited the analysis to one biological replicate per group, the sections cannot be treated as independent group replicates and no inferential statistical comparison was performed. These observations should therefore be interpreted as qualitative evidence consistent with, but not independently validating, the plasma cytokine findings of LPS-induced systemic inflammation. Cy7-tagged J-aggs were also visualized within cerebral vascular structures after perfusion (Fig. 11). PA data acquired using Cy7-tagged J-aggs were excluded because the fluorescently tagged formulation had a lower stock concentration than the standard formulation used for PA analysis (200 µM versus 400 µM).

**Fig. 10:**
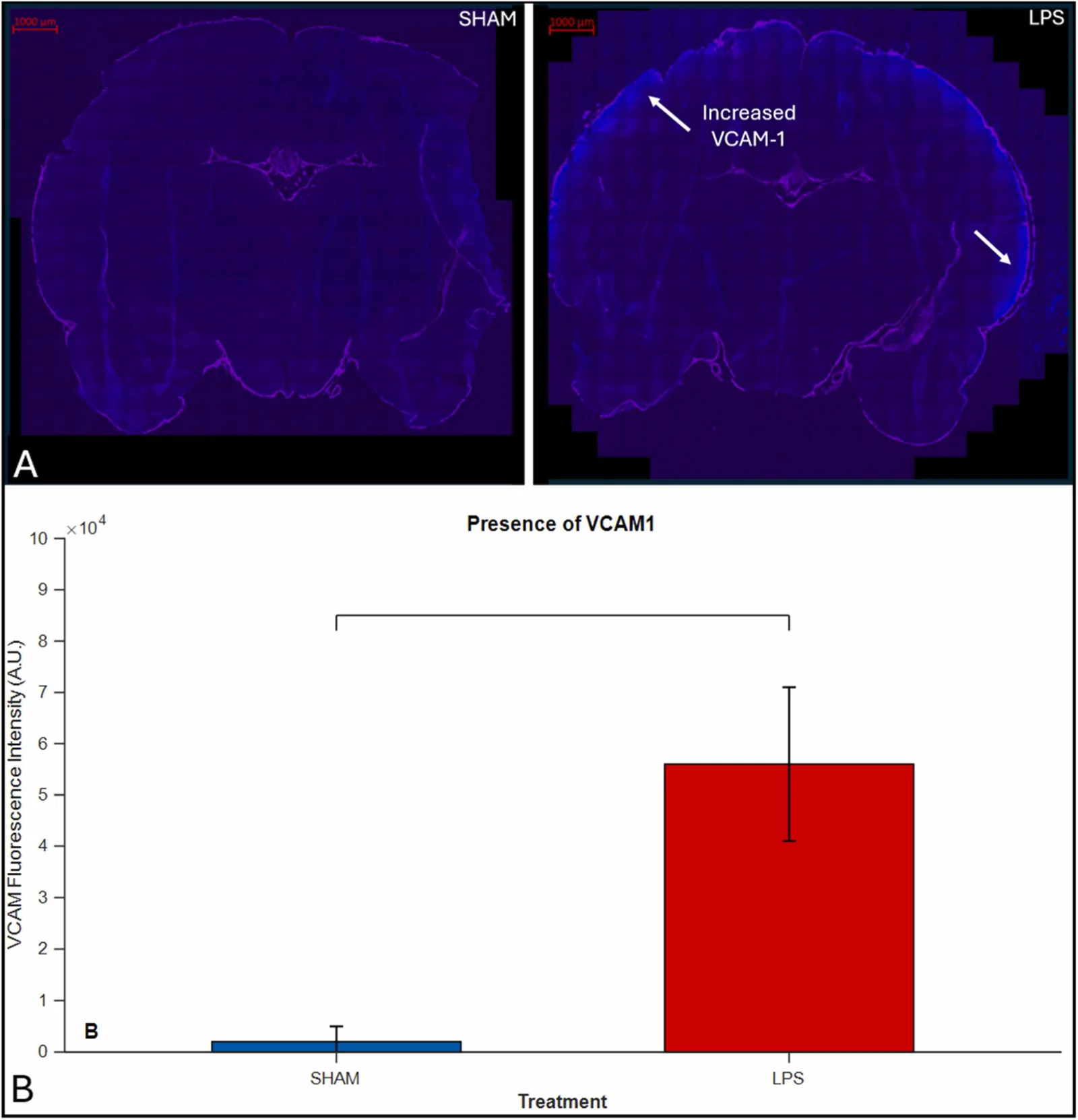
(A) Representative VCAM-1 immunofluorescence in brain tissue from one sham-treated and one LPS-treated rat. VCAM-1 is shown in blue and laminin in purple. Images were acquired using identical settings. Because only one animal per group was available, the images and section-level fluorescence measurements are illustrative and do not support group-level statistical inference. (B) Bar plot showing fluorescence corresponding to VCAM-1 expression for LPS and sham group. Three tissue sections were examined from one animal in each treatment group (sham n = 1, LPS n = 1). Due to limited sample size, VCAM-1 enhancement is representative/illustrative only rather than quantitative. Error bars represent one standard deviation of the mean of the three tissue sections used in each rat.

**Fig. 11:**
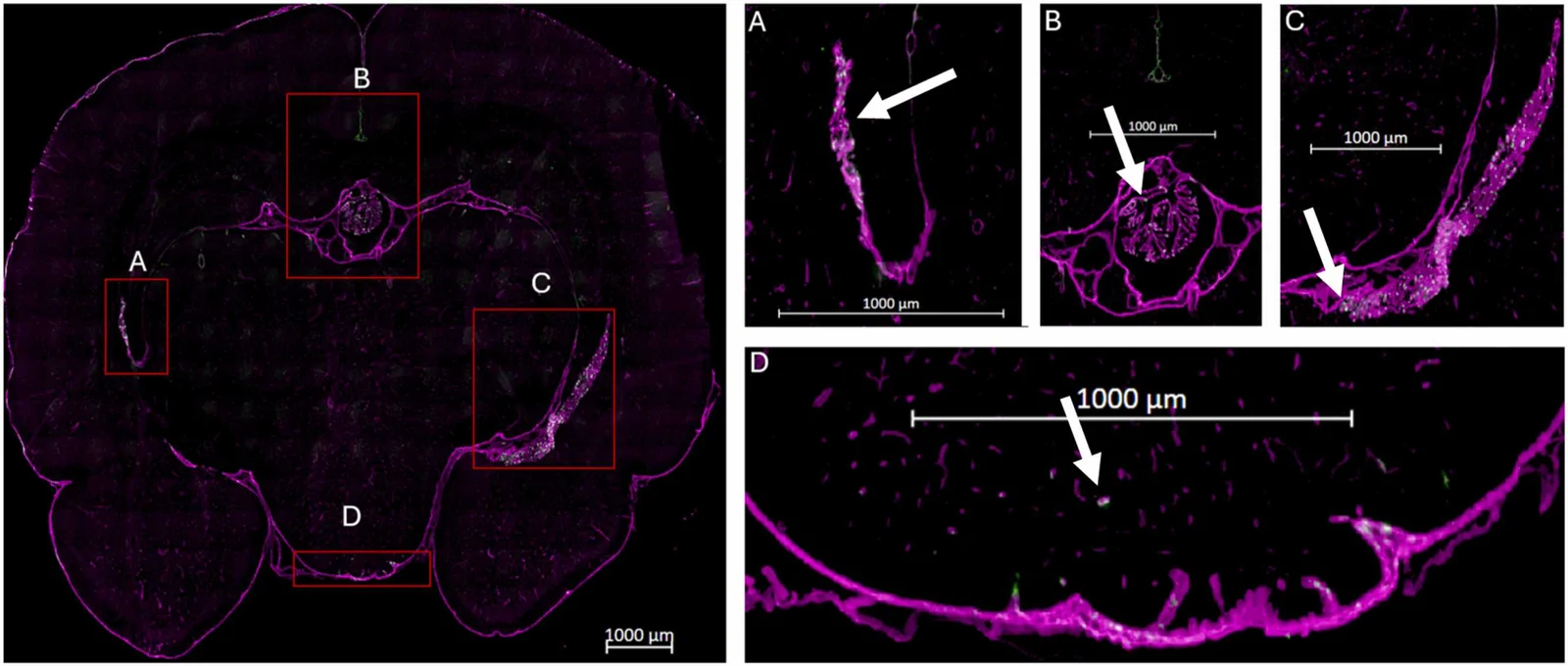
Representative localization of Cy7-tagged indocyanine green J-aggregates (J-aggs) in brain tissue from one sham-treated rat. Laminin is shown in purple and Cy7-associated fluorescence in green. White arrows indicate punctate Cy7 signal within laminin-positive vascular regions. Scale bar: 1 mm. Magnified views highlight region specific signals, including localized accumulation along vascular structures including the: A) lateral ventricle B) choroid plexus. C) corticofugal tract. D) microvasculature of the hypothalamus. Histological tissues were perfused and harvested following the 4-hour imaging time point. White arrows show punctate areas of Cy7 signal, corresponding to accumulation of J-aggs in cerebral vasculature.

## 4 Discussion

Contrast-enhanced transcranial PAI with J-aggs detected inflammation-associated changes in cerebral PA signal kinetics following systemic LPS administration in 3-week-old female rats. The largest divergence of PA signal between sham and LPS animals occurred 4 h post-treatment. Because peripheral biodistribution and cerebral perfusion were not independently measured, the observed difference cannot be assigned to a specific mechanism. Possible contributors of altered P90 include altered J-aggs circulation or cerebral delivery, inflammation-related changes in cerebral perfusion or microvascular function, and changes in the hemoglobin contribution to the 890 nm PA signal.

The present data directly support three principal findings: cerebral P90 increased at the imaging time point acquired approximately 5 min after IV J-aggs administration; the longitudinal P90 response differed between sham and LPS-treated animals from baseline to 4 h (group×time, p = 0.0262); and LPS induced systemic inflammatory activation, as indicated by elevated plasma cytokine concentrations. However, because the *in-vivo* data were acquired at a single wavelength of 890 nm, P90 represents a composite PA signal arising from J-aggs and endogenous absorbers, principally hemoglobin, rather than a J-aggs-specific measurement. The single-wavelength data also cannot determine whether partial conversion of J-aggs to monomeric or dimeric ICG occurred *in-vivo*. Altered tracer biodistribution and inflammation-related changes in cerebral perfusion, blood volume, or oxygenation therefore remain plausible explanations for the lower and more variable 4-hour P90 response in the LPS group, but these mechanisms were not independently measured. The present results should consequently be interpreted as pilot feasibility and hypothesis-generating evidence rather than as a mechanistic demonstration of altered J-aggs biodistribution.

In both sham and LPS groups, J-aggs injection increased cerebral ΔP90(%) to ~20–25%, suggesting successful vascular contrast enhancement following IV injection. At 2-hours post-treatment after the 2nd IV J-aggs injection, the sham group had a notable enhancement in ΔP90(%) (40% above baseline) compared to the LPS group (20%), however the difference was not statistically significant. At 4-hours following saline/LPS IP injection, the sham group exhibited significant enhancement (70% higher with respect to baseline) compared to the LPS group (30% higher with respect to baseline) resulting in a significant group × time interaction (p = 0.0262). This delayed divergence may reflect altered J-aggs circulation or cerebral delivery, inflammation-related hemodynamic changes, or a combination of these mechanisms. A Linear Mixed-Effects Model was used to account for repeated longitudinal measurements acquired from the same animals across imaging sessions.

Several non-exclusive mechanisms could contribute to the lower 4-hour P90 in the LPS group, each consistent with the data, but the data do not uniquely distinguish among them. Peripheral sequestration was not measured in the present study; therefore, the following mechanisms are hypotheses based on prior nanoparticle and inflammation literature rather than findings from the current dataset. First, increased peripheral clearance of J-aggs through inflamed, hyperpermeable endothelium in lung, liver, and spleen would reduce systemic availability and the flux of J-aggs through cerebral microvasculature [49]. One study reported that reduced Evans blue concentration in plasma following IV injection was observed in LPS-treated rats, likely due to extravasation [59]. Other studies have shown that nanoparticles have significantly increased retention in animals that received LPS in organs such as the spleen, liver, and lung; where enhanced uptake was a function of particle size [45]. Retention of nanoparticles in the spleen, liver, lung, or other organs could result in less nanoparticles available for circulation in cerebral vasculature.

Systemic inflammatory activation following LPS administration was demonstrated by the increased plasma concentrations of pro-inflammatory cytokines. In representative brain sections from one animal per group, stronger VCAM-1-associated fluorescence was observed in the LPS-treated specimen than in the sham specimen. (Fig. 10) However, this observation is qualitative and does not establish group-level upregulation of cerebral VCAM-1. Cerebrovascular inflammation following LPS exposure may alter cerebral perfusion and contribute to variability in PA signal intensity. Previous studies have reported leukocyte infiltration and capillary stalling within cerebral vessels after LPS administration [7,60,61], which could reduce local delivery of J-aggs and alter blood volume and oxygenation within affected microvessels. These vascular disturbances may contribute to the reduced visualization of deeper vascular structures, including the subependymal and lenticulostriate arteries, observed 4 h after LPS administration (Fig. 6), as well as the heterogeneous spatial distribution of P90 across animals. Systemic hemodynamic alterations induced by LPS, including hypotension, vasodilation, and altered cardiac index, may further influence PA measurements. However, because animals were imaged under 100% medical oxygen with isoflurane anesthesia, global oxygenation changes were likely minimized, with blood flow alterations expected to remain localized to regions affected by capillary stalls due to cerebral autoregulation.

Inflammation-related changes in functional cerebral blood volume may also affect the measured P90 independently of oxygenation. This effect may reflect a reduced perfused vessel density and reduced optical absorption within the imaging region. In a mouse PAI study that used a 5 mg/kg dose of IP LPS, the closest dose to the 3 mg/kg used here, normalized vessel intensity decreased by approximately 10% from baseline at four hours, while normalized vessel density and vessel-area ratio decreased by approximately 1.5% and 1.9%, respectively. [62] That study also reported that capillary-rich regions produced weaker PA signal than regions dominated by larger vessels despite similar vessel-area ratios. In the present study, J-aggs likely increased sensitivity to small vessels by increasing absorption at 890 nm before LPS-induced microvascular dysfunction was established. Thus, LPS-induced capillary stalling, reduced perfused vessel density, and/or redistribution of flow away from smaller vessels could reduce J-aggs delivery to the microvasculature and lower the P90 response. Cerebral microcirculation studies support this mechanism, showing that LPS increases the percentage and duration of capillary stalls and prolongs capillary transit time. [63] These effects could contribute to the attenuated enhancement observed in LPS animals, although they cannot be separated from altered systemic J-aggs circulation or endogenous hemoglobin-related signal without an LPS-only control or an independent perfusion measurement.

Another possible explanation for reduced J-aggs signal in the LPS group is increased sequestration and clearance of circulating particles by activated mononuclear phagocyte organs such as the liver and spleen, particularly through Kupffer cell activity [46,64,65]. This enhanced systemic clearance may reduce the circulating J-aggs pool available within cerebral vessels. This hypothesis could be evaluated using multispectral PA imaging to assess whether spectral shifts toward shorter wavelengths, consistent with J-aggs degradation to ICG, are present within abdominal organs.

The PA data findings are consistent with, but do not uniquely identify, a combination of reduced circulating J-aggs concentration (driven by peripheral extravasation and mononuclear phagocyte system sequestration) and altered intracerebral J-aggs distribution (driven by reduced blood flow and capillary stalling); LPS-induced changes in cerebral hemodynamics that alter the hemoglobin contribution to the 890 nm signal cannot be excluded as a contributor. J-aggs produced a marked enhancement of PA signal immediately following IV injection as seen in Fig. 8. Post-injection timepoints were not included in the statistical model, so this observation is descriptive. At 2-hours after IP LPS there were no significant differences between groups, which may be due to minimal systemic inflammatory effects that occurred between IP injection and 2-hours following IP injection. However, 4-hours after IP injection, there was a significant difference in P90 between groups, which could indicate that consequences of systemic inflammation have significantly increased, which is supported by the cytokine panel. Evidence of altered distribution of P90 between sham and LPS groups as seen in Fig. 6 supports the hypothesis that J-aggs distribution in cerebral vasculature may be altered due to reduced blood flow and capillary stalling. However, histology should be performed to validate narrowing of cerebral vessels and presence of leukocytes in cerebral capillaries. These mechanisms could also be investigated using cranial window intravital microscopy with fluorescently labeled J-aggs, however, maintaining physiological stability during imaging may be challenging due to the combined effects of anesthesia, LPS exposure, and surgical preparation.

In previous work, azide-stabilized J-aggs kinetics have been studied *in-vivo* in healthy mice (n = 2 female nude mice) [43] using PAI in abdominal organs such as the liver and spleen. PA data showed that J-aggs remained in circulation for up to 120 min after injection, with a circulation half-life 10 times longer than ICG. These prior observations informed the approximate 2-hour interval between doses, which was intended to permit a substantial decline in circulating J-aggs associated PA signal before administration of the second dose. However, the J-aggs used in previous work were surface modified to increase circulation half-life, thus the circulation half-life of the J-aggs reported here is likely reduced. J-aggs metabolism, blood concentration, organ uptake, and clearance were not measured directly in the present study. Consequently, the observed longitudinal PA changes cannot be used to estimate pharmacokinetic parameters or determine whether LPS altered J-aggs clearance. Dedicated future studies should include serial blood sampling and quantitative organ biodistribution after a single dose. The study examined baseline signal, 2-hours after IP saline/LPS, and 4-hours after IP saline/LPS to provide a gradual increase in inflammatory events with peak activity in reported cytokine markers such as TNF-α (first detectable at 2-hours with minimal activity at 4-hours after LPS injection) and IL-6 (peak activity at 2-hours after LPS injection with gradual decrease 4-hours onwards after LPS injection) [66]. Because all animals received two J-aggs injections, the 4-hour measurement reflects cumulative exposure and may include residual contrast from the first injection together with the circulation and clearance kinetics of the second injection. The present design therefore cannot determine whether a single J-aggs dose administered after establishment of the inflammatory response would have produced the same group difference.

As a preliminary step toward a complete (3D) multispectral acquisition dataset, 2D single-plane multispectral PA spectra were also collected at each timepoint prior to the primary 3D single-wavelength dataset (Supplemental Figures 4–5). As described in the Methods, the absence of fiducial-based repositioning resulted in a high degree of anatomical drift across the longitudinal imaging session, such that the whole-brain ROI used for spectral quantification did not consistently capture the same coronal plane over time. Given this, the multispectral PA data were not used to draw conclusions about J-aggs-specific spectral signal or its relationship to inflammation; rather, they reinforce the need for stable, co-registered 3D multispectral acquisition, and are included to inform the design of future longitudinal multispectral PA studies.

This study has several limitations. Firstly, there are a limited number of rats used in this pilot (n = 5 for sham, n = 6 for LPS). Because the study was not prospectively powered for definitive group comparisons, the observed effect estimates and p-values should be interpreted as exploratory and require confirmation in a larger, prospectively powered cohort. The findings presented in this study are specific to juvenile 3-week-old females, weighing 40–70 g with an average weight of ~50 g. At 3-weeks-old, rats remain developmentally distinct from adults [67]. Postnatal maturation involves continued remodeling of the cerebral microvascular network and maturation of interactions among endothelial cells, pericytes, vascular smooth muscle cells, astrocytic endfeet, and neurons within the neurovascular unit [68]. Regulation of cerebral blood flow also differs during development, including resting perfusion, pressure-dependent autoregulation, vascular reactivity to arterial CO_2_ and O_2_, and neurovascular coupling [69]. These developmental differences can affect cerebral blood volume, oxygen saturation, vessel caliber, and intravascular tracer transport, all of which may influence the measured PA amplitude. Age and sex can additionally modify responses to LPS, including systemic cytokine production and regional brain inflammatory markers [[70], [71], [72]]. The present findings should therefore be interpreted as specific to juvenile female rats until they are validated in adult animals of both sexes.

Anesthesia adds an additional source of physiological variability in the present study. All longitudinal acquisitions were performed under 2.5% isoflurane, which standardized the nominal imaging conditions across treatment groups but does not ensure identical cerebrovascular or blood–brain barrier responses among animals. Using multidimensional optical imaging, Li et al. reported that isoflurane induced hemispheric differences in blood-brain barrier permeability and associated vascular microstructure and function [73]. Although their imaging platform and experimental objectives differed from those in this study, their findings demonstrate that anesthetic state can influence blood-brain barrier associated transport and vascular optical signals. This is relevant because the measured P90 at 890 nm contains contributions from both IV J-aggs and endogenous hemoglobin. Anesthesia-related changes in vascular tone, cerebral blood volume, oxygenation, or tracer transport could therefore contribute to P90 variability or interact with LPS-induced physiological changes. Maintaining the same anesthetic protocol across groups reduces systematic experimental bias but does not exclude treatment × anesthesia interactions. Future studies should therefore record anesthetic depth and systemic physiological variables continuously and, where technically feasible, compare results with awake acquisitions.

Next, the data acquisition in this study used a single PA wavelength corresponding to the peak PA response of the J-aggs formulation, λ = 890 nm. At 890 nm, hemoglobin absorption is non-negligible and cannot be spectrally separated from the J-aggs contribution; consequently, the observed P90 changes reflect a composite signal from both J-aggs and circulating hemoglobin rather than a J-aggs specific measurement. Because P90 emphasizes the brightest voxels within the cerebral ROI, it may be disproportionately influenced by large vessels, local perfusion differences, and variation in the spatial distribution of high-amplitude voxels. P90 should therefore be interpreted as a summary measure of high-intensity cerebral PA signal rather than as a vessel-size-independent or tracer-specific metric.

LPS-induced hemodynamic changes such as hypotension, altered cardiac output, shifts in cerebral blood volume and oxygen saturation, and fluid accumulation due to induction of anesthesia and/or LPS, can therefore alter the measured PA signal independently of J-aggs distribution. The pre-J-aggs baseline P90 difference between groups (p = 0.0659) is consistent with such a non-J-aggs contribution to signal variability between cohorts. Multispectral 3D acquisition would be required to separate these components more directly. However, stable 3D multi-wavelength acquisition was not technically feasible with the software version available during this study. Attempts to initiate multi-wavelength 3D sweeps resulted in system-level acquisition errors requiring restart. Although sequential single-wavelength 3D scans at multiple wavelengths could theoretically have been acquired, this approach would have introduced substantial risk of respiratory motion, transducer–animal repositioning, coupling changes, inconsistent timing between wavelengths, and scan-to-scan variation, making spectral unmixing unreliable. Future studies should therefore use stable co-registered 3D multispectral acquisition across wavelengths selected to sample deoxyhemoglobin, oxyhemoglobin, ICG features, and the J-aggs absorption peak at 890 nm. Pre-contrast baseline scans, post-contrast longitudinal scans, spectral unmixing using VevoLab or custom open-source algorithms, and fluence or spectral-coloring correction would help estimate the relative contributions of deoxygenated hemoglobin, oxygenated hemoglobin, and J-aggs. These approaches should be paired with direct hemodynamic monitoring, blood oxygenation measurements, and ex vivo biodistribution validation to determine whether LPS-associated P90 changes are driven primarily by altered J-aggs kinetics, cerebral blood volume, oxygenation, or a combination of these factors.

A limitation of this study is the absence of a negative control cohort receiving IP LPS or saline without IV J-aggs. Such a comparison was not included because pre-contrast PA signal in the cerebral vasculature was not expected to differ meaningfully between groups: endogenous hemoglobin absorption alone produces low signal-to-noise contrast for the small and deep cerebral vessels of interest here, such as the subependymal and lenticulostriate arteries, which are difficult to resolve from background in the absence of an exogenous contrast agent. This is consistent with prior work showing that pre-contrast PA images of mouse brain vasculature exhibit weak, low-SNR signal limited to large superficial vessels such as the middle cerebral artery, with small vessels becoming clearly resolvable only after administration of an exogenous contrast agent [29]. Similarly, in the rat brain, PEGylated gold nanoshells with a NIR-I absorption peak at 800 nm produced a gradual enhancement of PA signal in the cerebral vasculature of up to 63% across sequential contrast agent administrations, comparable to the wavelength range and IV administration route used for J-aggs here [74]. Without J-aggs, we would therefore expect the resulting baseline PA data to have a high level of noise relative to any inflammation-related signal, making it difficult to reliably distinguish group differences related to LPS-induced changes from measurement variability at the sample sizes used in this pilot study. J-aggs were therefore considered necessary to generate sufficient contrast in small and deep cerebral vessels to detect inflammation-associated changes in this model. Future work should include a vehicle-only (no J-aggs) negative control cohort, powered with a larger number of animals, to determine whether, and to what extent, LPS alters cerebral PA signal in the absence of J-aggs. To inform the design of these future vehicle-control cohorts, we performed a prospective power analysis across assumed differences in cerebral PA signal (Supplemental Figure 6). Guevara et al. reported an approximately 10% reduction in locoregional cerebral oxygen saturation following LPS-induced inflammatory injury in neonatal rats [58], supporting the possibility that inflammation-related oxygenation changes could contribute to cerebral PA measurements. Based on this prior observation, an assumed 5% between-group difference in PA signal was included as an illustrative small endogenous contribution. Under the statistical assumptions used, substantially more than six animals per group would be required to detect an effect of this magnitude with 80% power. The 5% PA-signal difference is a modelling assumption rather than a direct conversion of oxygen saturation to PA amplitude and does not indicate that such a change occurred in the present study.

Immunohistochemistry was limited to one animal per treatment group due to tissue loss during freezing and sectioning. The three sections examined from each animal were within-animal technical observations and did not provide independent biological replication. Consequently, the VCAM-1 images are descriptive and cannot statistically validate a treatment-related difference in cerebral inflammation. Similarly, Cy7-tagged J-aggs were visualized only in a representative sham specimen; these images provide spatial evidence of J-aggs-associated fluorescence within cerebral vascular structures but cannot establish treatment-dependent differences in localization.

Females were used exclusively in this work to reduce variability in data due to sex differences. Future studies should include male rats to examine sex differences in inflammatory and immune response to LPS and the impact on the PA signal. Young rats were used due to their thin cranial bones allowing for NIR PA imaging at high frequencies. PA imaging in adult rats is challenging due to high optical and acoustic scattering, leading to severely degraded image quality, increased artifacts, and decreased SNR for deeper tissues. One potential translational route is imaging through the neonatal fontanelle, which may reduce skull-related optical and acoustic attenuation. However, feasibility, safety, achievable imaging depth, and diagnostic performance would require dedicated human-specific validation.

Some variability in animal P90 data could be due to induction of anesthesia which alters respiratory rate and hemodynamics, dilates blood vessels, alters core temperature regulation, and is an additional stressor. Although the stereotactic holder, volumetric acquisition, and landmark-based plane selection reduced sensitivity to single-plane repositioning error, no fiducial markers or formal image-registration algorithm were used. Thus, residual differences in probe angle, acoustic coupling, and position may therefore have contributed to variability in P90 measurements. Optical attenuation due to high concentration of J-aggs in superficial vessels could reduce fluence to deeper vessels, limiting PA signal at depth and influence P90 pixel selection.

The radiant exposure at the laser fiber surfaces was approximately 178 mJ/cm² per illumination arm, roughly 3.7-fold higher than the ANSI Z136.1 single-pulse maximum permissible exposure for skin at 890 nm, which is approximately 48 mJ/cm² for a nanosecond pulse, calculated as 20·$C_{A}$ with $C_{A}$≈ 2.4 [75,76]. Because illumination was delivered as a 20 Hz pulse train, the ANSI Z136.1 multiple-pulse criterion must also be considered. Assuming an upper bound of approximately 4 pulses delivered to a single surface site during the translated 3D sweep, the corrected per-pulse exposure limit is approximately 34 mJ/cm², corresponding to an approximately 5.2-fold exceedance at the fiber surface. Although optical attenuation by the scalp, skull, and intervening tissues substantially reduces the fluence reaching the brain, this attenuation does not remove the requirement to maintain exposure at the skin surface within applicable safety limits. Translation to human studies may be challenging with the current imaging setup, as high-frequency US exhibits high acoustic attenuation, limiting measurements to very superficial structures while providing high spatial resolution. However, with a lower frequency probe, PAI through the fontanelle in pediatric patients may be possible to a depth of ~2 cm at the cost of imaging resolution [77]. A clinically realistic system would require ANSI-compliant illumination, direct surface dosimetry, broader or multi-angle light delivery, improved optical coupling and detector sensitivity, lower-frequency acoustic arrays, and quantitative modelling of light and acoustic propagation at the intended imaging depth.

In the future, multiple sizes of J-aggs will be investigated to examine differences in PA signal time-kinetics between treatment groups. This study used 1 micron J-aggs; however, J-aggs are size-tunable and can be manufactured between 100 and 1000 + nm [78]. Smaller J-aggs (e.g., 200 nm) may exhibit different biodistribution kinetics, compartmentalizing at different rates in LPS-treated groups, and may exhibit altered half-lives in healthy animals due to their altered surface area. Future work should combine multispectral transcranial PAI with ex-vivo and *in-vivo* biodistribution validation, including fluorescently tagged J-aggs or tomographic PA imaging of peripheral organs such as the liver, spleen, lung, and kidneys. These experiments would help determine whether reduced cerebral PA enhancement in LPS-treated animals is associated with altered systemic clearance, peripheral sequestration, reduced cerebral delivery, or degradation of J-aggs into shorter-wavelength ICG-associated species. Future studies should incorporate an independent measure of cerebral perfusion, such as 3D Doppler-based flow imaging or contrast-enhanced ultrasound with microbubbles, to help distinguish altered tracer kinetics from LPS-induced hemodynamic changes.

In this pilot study, transcranial contrast-enhanced PAI with non-encapsulated J-aggs detected a significant group × time difference in cerebral P90 4-hours post-LPS, supporting further preclinical evaluation of this approach for monitoring inflammation-associated cerebral PA signal changes. Larger studies across both sexes and multiple wavelengths, together with improved physiological monitoring and exposure conditions compatible with established safety limits, are required before translational potential can be assessed.

## 5 Conclusion

This pilot study demonstrated that transcranial contrast-enhanced PAI using non-encapsulated ICG J-aggs can detect longitudinal differences in composite cerebral PA signal in a 3-week-old rat model of systemic inflammation. IV administration of J-aggs was followed by measurable enhancement of composite cerebral PA signal in both sham and LPS-treated animals, consistent with transcranial contrast enhancement following J-aggs administration. However, signal kinetics diverged between groups over time, with sham animals exhibiting sustained enhancement and LPS-treated animals showing attenuated and variable enhancement 4 h after IP LPS administration. This resulted in a significant group × time interaction that may reflect altered J-aggs circulation or cerebral delivery, inflammation-related changes in cerebral blood volume or oxygenation, or a combination of these mechanisms. Plasma cytokine measurements independently confirmed systemic inflammatory activation in the LPS-treated group but did not validate the mechanism underlying the cerebral PA signal difference. Together, these results support further preclinical evaluation of contrast-enhanced transcranial PAI for monitoring inflammation-associated cerebral PA signal changes. Multispectral validation, larger cohorts, and exposure conditions compatible with established safety limits will be required before clinical or bedside applicability can be assessed.

## CRediT authorship contribution statement

**Filip J. Bodera:** Writing – review & editing, Writing – original draft, Visualization, Validation, Software, Resources, Project administration, Methodology, Investigation, Funding acquisition, Formal analysis, Data curation, Conceptualization. **Elizabeth Berndl:** Writing – review & editing, Resources, Project administration, Methodology, Formal analysis, Data curation. **Shrishti Singh:** Writing – review & editing, Validation, Investigation, Conceptualization. **Anthony Tay:** Writing – review & editing, Resources, Project administration, Data curation. **Greeshma Tarimala:** Writing – review & editing, Resources, Data curation. **Samuel K. Jensen:** Writing – review & editing, Resources, Methodology, Investigation, Conceptualization. **Parag V. Chitnis:** Writing – review & editing, Validation, Supervision, Resources, Project administration, Methodology, Investigation, Funding acquisition, Conceptualization. **Remi Veneziano:** Writing – review & editing, Validation, Supervision, Resources, Methodology, Investigation, Funding acquisition, Formal analysis, Conceptualization. **Mark J. McVey:** Writing – review & editing, Writing – original draft, Supervision, Methodology, Investigation, Funding acquisition, Formal analysis, Conceptualization. **Michael C. Kolios:** Writing – review & editing, Writing – original draft, Supervision, Project administration, Funding acquisition, Formal analysis, Conceptualization.

## Ethics declaration

This study was conducted in accordance with the ARRIVE (Animal Research: Reporting of In Vivo Experiments) guidelines. This study was approved by the St. Michael's Hospital. (Approval No. 220921)

## Declaration of Generative AI and AI-assisted Technologies in the Manuscript Preparation Process

During the preparation of this work the authors used ChatGPT 5.5 to improve clarity in parts of the introduction and discussion; and Claude Sonnet 4.6 with adaptive thinking to assist in generation of MATLAB code for statistical tests. After using these services, the authors reviewed and edited the content as needed and take full responsibility for the content of the published article.

## Declaration of Competing Interest

The authors declare that they have no known competing financial interests or personal relationships that could have appeared to influence the work reported in this paper.

## Data Availability

Data will be made available on request.
