# Supplementary material for: Transcranial photoacoustic imaging of indocyanine green J-aggregates reveals cerebral signal changes in lipopolysaccharide-induced systemic inflammation
Source: Photoacoustics. 2026 Sep 22;51:100885. doi: 10.1016/j.pacs.2026.100885 (PMC13635703; doi:10.1016/j.pacs.2026.100885)
Supplement: Supplementary file 1 [file mmc1.docx]

Supplemental Figures

Supplemental Figure 1: (A) Measured values are plotted against in-sample, conditional model-predicted values of percent change from baseline. The solid red line denotes the linear regression, and the dotted line represents the identity line. Agreement was quantified by root mean square error (RMSE=16.56), mean absolute error (MAE=12.50), and Bland-Altman bias (=0.0003) suggesting moderate variability in model fit with no net over- or under-prediction on average; as shown in panel (B), however, this near-zero average bias masks a magnitude-dependent bias. However, the 95% limits of agreement were wide (-33.23 to +33.23 percentage points), indicating that for any given individual, response may differ from conditional predicted value by up to 33% in either direction. A leave-one-animal-out (LOAO) RMSE (17.34) and MAE (12.97) are reported as animal-level cross-validated error estimates. (B) The Bland-Altman proportional bias analysis showed a positive slope (0.32, 95% CI [0.09, 0.55], p = 0.008), indicating that prediction error increased with the magnitude of the response being predicted.

Supplemental Figure 2: Normal quantile plot (top) with 95% confidence intervals and distribution histogram (bottom). Standardized residuals are plotted against theoretical normal quantiles; the solid red line is the reference line and dashed curves are pointwise 95% confidence bands. Most residuals lie along the reference line within the central range, while approximately 15% of residuals fell outside the 95% confidence bands, with deviations concentrated in the tails, suggesting heavier-tailed residual behaviour. To assess robustness to this tail behaviour, the linear mixed effects model was refit using subject-level bootstrap resampling; animals were resampled with replacement within treatment group, with all repeated measurements from each selected animal retained to preserve the within-animal longitudinal structure. The same linear mixed effects model structure was refit for each of 1000 bootstrap samples, and percentile bootstrap 95% confidence intervals were calculated from the resulting contrast-estimate distributions. Because resampling was performed over a small number of clusters (sham n=5, LPS n=6), these intervals should be interpreted as approximate. For the primary top 10% pixel metric, the baseline - 4 hours group × timepoint contrast remained negative, with an estimate of −41.7% and a bootstrap 95% CI of −68.5% to −16.1%.

Supplemental Figure 3: Sensitivity analysis to determine change in effect size based on top percentage of pixels using linear mixed effects (LME) test. (A) LME p-values for predefined group × timepoint contrasts plotted across top-pixel thresholds. The P90 metric, corresponding to the top 10% of pixels, was selected a priori based on prior measurements; this threshold sweep was performed to assess robustness rather than to select the analysis threshold. The baseline - 4 hour contrast remained below p = 0.05 from the top 5% to top 20% pixel thresholds, with p = 0.054 at the top 25% threshold, whereas the baseline - 2 hours and 2 hour - 4 hour contrasts remained above p = 0.05 across all thresholds. (B) Corresponding group × timepoint contrast estimates with subject-level bootstrap 95% confidence intervals. The baseline - 4 hour contrast remained consistently negative across thresholds, ranging from −46.3% to −35.7%, with bootstrap 95% confidence intervals excluding zero at all thresholds, including the top 25% threshold at which the linear mixed effects p-value was 0.054; the two inference approaches therefore diverge marginally at the lowest threshold tested. In contrast, the baseline - 2 hour and 2 hour - 4 hour contrasts had confidence intervals that crossed zero across all thresholds. These results indicate that the primary baseline - 4 hour effect was stable across the tested top-pixel thresholds, while lower thresholds produced modest attenuation of the estimated effect, consistent with inclusion of additional lower intensity pixels and/or reduced spatial extent of PA signal.

Supplemental Figure 4: Longitudinal B-mode ultrasound and photoacoustic imaging of rat brain following intravenous (IV) administration of indocyanine green J-aggregates (J-aggs). (A–E) B-mode ultrasound images acquired at baseline, +10 minutes after intraperitoneal (IP) injection of saline (+ IV J-aggs), +2 hours after IP, +2 hours + 10 minutes after IP (+ IV J-aggs), and +4 hours after IP. (F–J) No imaging parameters were changed between timepoints and no fluence or compensation gain was applied. The animal shown is from the SHAM group (ID = R5). The corresponding B-mode images with the whole-brain region of interest (ROI, green outline) delineated for signal quantification. (K–O) Photoacoustic (PA) mode images acquired at 890 nm across the same timepoints, displayed on an amplitude scale of 0–30 A.U. (P–T) PA images with the whole-brain ROI (green outline) superimposed for quantification of intracranial signal. Comparison of the B-mode anatomy across timepoints (A–E, F–J) reveals that the same coronal plane and anatomical landmarks were not consistently reacquired at each imaging session, with visible shifts in probe position and slice depth relative to the brain over time. This anatomical drift means the whole-brain ROI does not correspond to an identical brain region across timepoints, introducing a confound for longitudinal multispectral photoacoustic (MSPA) quantification and underscoring the need for 3D multispectral data, and improved co-registration or fiducial-based repositioning in future imaging sessions.

Supplemental Figure 5: Energy-normalized photoacoustic (PA) spectra of whole-brain signal in sham and LPS-treated animals across the imaging time course. Qualitatively, the spectra acquired after intravenous (IV) indocyanine green J-aggregates (J-aggs) administration retained a broad longer-wavelength feature near 880–910 nm, without an obvious isolated relative increase centered in either the approximate 715–725 nm ICG dimer region or the 780–800 nm ICG monomer region; this observation is descriptive only and does not establish J-aggs concentration or exclude partial conversion to monomeric or dimeric ICG. Mean energy-normalized PA signal amplitude (A.U.) as a function of wavelength (680–970 nm) for sham (black) and LPS (red) groups, shown at baseline, +10 minutes after IP (+ IV J-aggs), 2-hour after IP, 2 hours +10 minutes after IP (+ IV J-aggs), and +4 hours after IP. Error bars denote ±1 SD across animals (sham n=5, LPS n=6). Apparent differences between sham and LPS spectra at each timepoint, as well as shifts in spectral shape and amplitude across timepoints within each group, should be interpreted with caution given the anatomical drift documented in the corresponding B-mode imaging. Because the whole-brain ROI did not consistently capture the same coronal plane and landmarks across sessions, group- and time-dependent differences in normalized PA signal cannot be distinguished from variability in probe positioning and slice registration, genuine differences in J-aggs concentration, or underlying physiological changes between groups. Furthermore, energy normalization corrects pulse-to-pulse laser energy only and does not correct wavelength-dependent attenuation of overlying tissue, so apparent changes in spectral shape may reflect spectral colouring rather than agent concentration.

Supplemental Figure 6: Prospective power analysis for future vehicle-control experiments. (A) Estimated number of animals required per group to achieve 80% power across a range of assumed between-group differences in cerebral photoacoustic signal in a two-sample two-sided t-test via the noncentral t-distribution, assuming a within-group standard deviation of 0.3 and α = 0.05. This two-sample framework is a simplification of the linear mixed effects analysis used in the present study and is likely to be conservative relative to a repeated-measures design with within-animal baseline normalization. (B) Estimated statistical power achievable with n = 6 animals per group across the same assumed effect sizes. The vertical orange line indicates an assumed 5% PA-signal difference (n = 64), included as an illustrative small agent-independent effect that could arise from endogenous hemodynamic changes. This scenario was informed by a previous neonatal rat study reporting an approximately 10% reduction in locoregional cerebral oxygen saturation following LPS-induced inflammatory injury [58]. The 5% PA-signal difference is a modelling assumption and is not a direct conversion of oxygen saturation to PA amplitude. The dashed horizontal line indicates n=6 animals per group in panel (A) and 80% power in panel (B).
